# Expanding the Toolbox for Label-Free Enzyme Assays: A Dinuclear Platinum(II) Complex/DNA Ensemble with Switchable Near-IR Emission

**DOI:** 10.3390/molecules24234390

**Published:** 2019-12-01

**Authors:** Moustafa T. Gabr, F. Christopher Pigge

**Affiliations:** Department of Chemistry, University of Iowa, Iowa City, IA 52242, USA; gabr@stanford.edu

**Keywords:** label-free assay, near-infrared, deoxyribonuclease I, platinum, aggregation-induced emission, supramolecular chemistry

## Abstract

Switchable luminescent bioprobes whose emission can be turned on as a function of specific enzymatic activity are emerging as important tools in chemical biology. We report a promising platform for the development of label-free and continuous enzymatic assays in high-throughput mode based on the reversible solvent-induced self-assembly of a neutral dinuclear Pt(II) complex. To demonstrate the utility of this strategy, the switchable luminescence of a dinuclear Pt(II) complex was utilized in developing an experimentally simple, fast (10 min), low cost, and label-free turn-on luminescence assay for the endonuclease enzyme DNAse I. The complex displays a near-IR (NIR) aggregation-induced emission at 785 nm in aqueous solution that is completely quenched upon binding to G-quadruplex DNA from the human c-myc oncogene. Luminescence is restored upon DNA degradation elicited by exposure to DNAse I. Correlation between near-IR luminescence intensity and DNAse I concentration in human serum samples allows for fast and label-free detection of DNAse I down to 0.002 U/mL. The Pt(II) complex/DNA assembly is also effective for identification of DNAse I inhibitors, and assays can be performed in multiwell plates compatible with high-throughput screening. The combination of sensitivity, speed, convenience, and cost render this method superior to all other reported luminescence-based DNAse I assays. The versatile response of the Pt(II) complex to DNA structures promises broad potential applications in developing real-time and label-free assays for other nucleases as well as enzymes that regulate DNA topology.

## 1. Introduction

Developing luminescent probes for rapid detection of biomolecules and/or monitoring of biochemical processes is an important objective in contemporary bio-organic chemistry [1,2,3,4,5]. Such probes can provide fundamental insight into mechanistic features of cellular events or function as diagnostic agents in biomedical applications. Additionally, probes for specific biocatalytic transformations are important bioanalytical tools for detection and quantification of enzymatic activity [6,7,8]. Significantly, elevated or reduced levels of specific enzyme activity often serve as biomarkers of human disease.

Deoxyribonuclease I (DNAse I) is the most abundant nuclease in human blood plasma. It is a non-restriction endonuclease that cleaves phosphodiester linkages within polynucleotide chains to release shorter oligonucleotides [9,10,11,12]. DNAse I functions as a waste-management nuclease through degradation of circulating DNA released into human serum upon cellular death [13]. Clinically, DNAse I may also serve as a functional biomarker in monitoring the progression of different human diseases [14,15,16,17,18]. For example, low DNAse activity in blood plasma of prostate cancer patients in comparison to healthy controls was demonstrated [14]. Recently, low urinary DNAse I level was identified as a marker for progression of lupus nephritis [15]. In addition, elevated serum DNAse I activity is a valuable marker of acute myocardial infarction and transient myocardial ischemia [16]. The correlation between serum DNAse I activity and immunoserological markers in systemic lupus erythematosus (SLE) patients has been advanced as a means of monitoring SLE progression [17].

Conventional methods for assessing DNAse I activity include enzyme-linked immunosorbent assays (ELISAs) [10], single radial enzyme-diffusion methods [19], and electrochemical assays [20]. These methods are time-consuming, labor intensive, and/or require use of covalently labelled DNA. These limitations are partially addressed by luminescence-based DNAse I assays that feature simplicity, sensitivity and ease of operation [21,22,23]. However, these methods often rely on the use of fluorophore-labelled DNA [24,25,26,27]. The high cost and synthetic challenges encountered in developing fluorophore-labelled DNA probes render label-free assays more convenient and cost-effective alternatives. Most existing label-free DNAse I assays are based on “turn-off” fluorescence signaling, which can lead to reduced sensitivity and false positive responses [28,29,30,31,32,33]. Consequently, “turn-on” luminescence bioassays are more attractive, but only one label-free turn-on DNAse I assay has been reported. This system, based on DNA-Ag nanocluster composites with graphene oxide, requires multiple steps and prolonged reaction times that restrict its potential utility, especially in high-throughput assays for DNAse I activity [23]. To the best of our knowledge, a sensitive, facile, multiwell-based and label-free luminescent DNAse I assay has not been developed (Table S1).

Luminescent platinum(II) complexes display intriguing photophysical properties and are attracting increasing interest in materials chemistry and optoelectronics [34,35,36,37,38]. In addition, the d^8^ electronic configuration and typical square planar coordination geometry observed in these complexes imparts a tendency to display metal-metal and/or π-π stacking interactions upon self-assembly [34,35]. These self-assembly events are often signaled by drastic color changes in the visible region and emergence of near-infrared (NIR) luminescence. This general emission profile has resulted in the use of several Pt(II) complexes as components in luminescent sensors for applications in materials chemistry and, less commonly, as biological probes [39,40,41,42,43,44,45].

**Figure 1:**
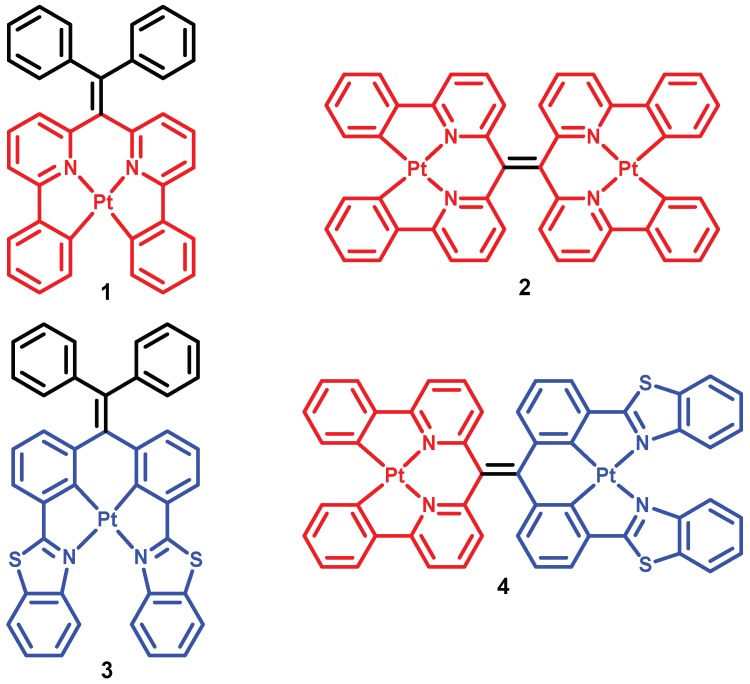
Structures of Pt(II) complexes **1**–**4**.

We recently reported rhenium(I) and platinum(II) complexes of tetraarylethylenes as luminescent probes for biomacromolecules and mismatched DNA, respectively [46,47,48,49]. As part of these efforts it was observed that cyclometalated Pt(II) complex **1** (Figure 1) displays significantly red-shifted emission in 9:1 Tris buffer:CH_3_CN solution compared to pure CH_3_CN (λ_em_ = 594 and 505 nm, respectively) [48].Similarly, bis(benzothiazole)Pt(II) complex **3** also was found to exhibit a slightly red-shifted emission in aqueous solution compared to emission in DMSO (Figure S9). We speculate that these luminescent properties may be affected by Pt···Pt interactions. In this work we have prepared bimetallic Pt(II) complexes **2** and **4** to test the hypothesis that introduction of additional Pt centers into the tetraarylethylene scaffold will further enhance the likelihood of metal-metal interactions upon aggregation-induced self-assembly, in turn resulting in even further bathochromic shifts in emission into the NIR region. Ultimately, we aim to harness this emission response through development of new probes for biomolecules and biomolecular processes. Toward this end, we have found that bis(platinum) complex **4** does indeed exhibit the targeted emission profile, and we have successfully exploited the switchability of NIR emission in **4** in the presence of DNA oligomers to develop an experimentally simple, sensitive, and label-free turn-on assay for DNAse I activity.

## 2. Results and Discussion

The synthesis of **2**–**4** was accomplished in high yield using a route similar to that previously reported for the synthesis of **1**. All new compounds were characterized by NMR and mass spectrometry, and UV–visible spectra of new complexes and their metal-free precursors were obtained (see Supporting Information and Figures S1–S7). Complex **2**, however, displayed absorbance (λ_abs_ = 416 nm, Figure S3) and emission (λ_em_ = 591 nm, Figure S8) spectra very similar to those obtained for mono-platinum complex **1**, and the wavelength of emission was unaffected by solvent-induced aggregation (Figure S8). 

**Figure 2:**
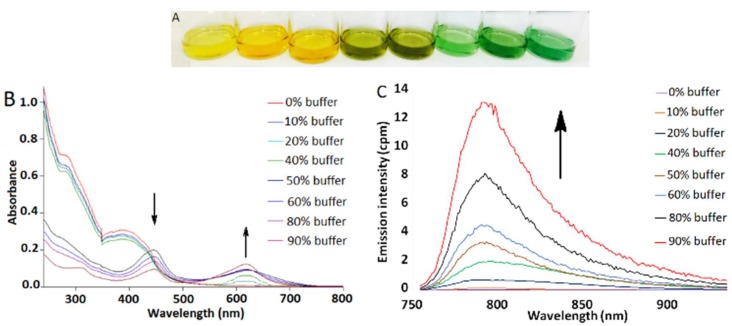
(**A**) Solution of **4** in DMSO/Tris buffer mixtures, [**4**] = 50 μM. Tris buffer percentage from left to right: 0, 10, 20, 40, 60, 70, 80, 90%. (**B**) UV–vis absorption changes of **4** in DMSO/buffer mixtures, [**4**] = 4 μM. (**C**) Emission spectra of **4** in DMSO/buffer mixtures, λ_ex_ = 445 nm, [**4**] = 4 μM. All experiments were performed at room temperature.

In contrast, complex **4** exhibited significant aggregation-induced bathochromic shifts in both absorbance and luminescence spectra. The UV–visible spectrum of **4** in DMSO shows a high-energy absorption band at 291 nm and a low-energy absorption band at 397 nm (Figure S7). However, incremental addition of Tris buffer (pH 7.5) to **4** resulted in dramatic changes in the color of the solution from yellow to green (Figure 2A) as well as in the appearance of a new absorption band at 621 nm (Figure 2B). Concomitant with UV–vis absorption changes, gradual emergence of a NIR emission band at 785 nm was also detected in the luminescence spectrum (Figure 2C). These spectral changes are attributed to aggregation of **4** via metal-metal and/or π-π stacking interactions, as has been previously reported for other platinum(II) complexes [34,35,39,40,41,42,43,44,45]. The self-assembly of **4** in 9:1 Tris buffer:DMSO was further confirmed by detection of nanoaggregates of 54.1 ± 2.7 nm size by dynamic light scattering (DLS, Figure S10). Significant signal broadening in ^1^H NMR spectra as a function of solvent was observed as well (Figure S11). The solvent-induced aggregation of **4** is further supported by a concentration-dependent UV–vis absorption study that shows deviation from Beer’s law for the absorption band at 621 nm (Figure S12). Finally, the disassembly of **4** as a function of increasing temperature resulted in significant attenuation of the NIR emission band at 785 nm, consistent with an emission signal that arises from intermolecular aggregation (Figure S13). 

**Figure 3:**
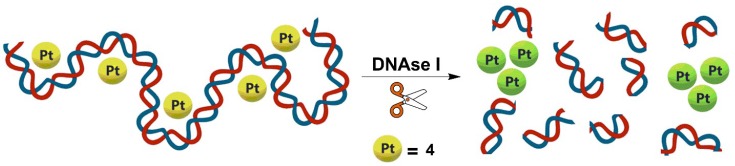
Schematic illustration of the proposed DNAse I assay through self-assembly of **4** in aqueous buffer upon DNA cleavage.

Given the ability of (tetraarylethylene)Pt(II) complexes such as **1** to bind DNA structures [48], we envisioned that potential non-covalent interaction of **4** with DNA oligomers would result in de-aggregation and shielding of the dinuclear platinum(II) complex from the aqueous environment, effectively quenching the NIR emission. Subsequent DNA cleavage by DNAse I would then release **4** back into the aqueous buffer resulting in recovery (turn-on) of NIR luminescence (Figure 3). The dependence of NIR emission of **4**/DNA ensembles on the DNA cleavage process would enable a label-free and turn-on assay of DNAse I activity.

**Figure 4:**
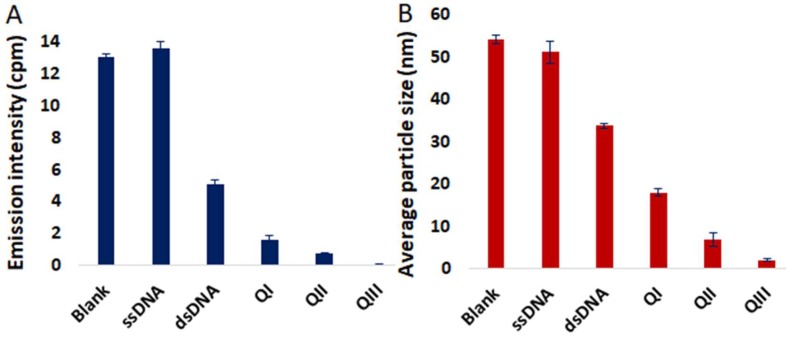
(**A**) Emission intensities of 4 at 785 nm in the absence and in the presence of ssDNA, dsDNA, **QI**, **QII** and **QIII**; λ_ex_ = 445 nm. (**B**) Average particle size measured by DLS of **4** in the absence and in the presence of ssDNA, dsDNA, **QI**, **QII** and **QIII**. [**4**] = 4 µM, [DNA] = 8 µM. Error bars represent standard deviation (*n* = 3).

Accordingly, the luminescence response of **4** in 9:1 Tris buffer:DMSO in the presence of various DNA structures was examined (Figure 4A and Figures S14–S17). Minimal change in the emission profile of **4** was observed in the presence of 24-mer single-stranded DNA (ssDNA) as demonstrated in Figure S15. However, considerable attenuation of the NIR emission band of **4** was achieved in the presence of a 12 base pair double-stranded DNA (dsDNA). Various metal complexes are known to bind to G-quadruplex DNA (QDNA) [50,51,52], and QDNA structures derived from three different oligonucleotides also were investigated for their effect on NIR luminescence of **4**. A bimolecular G-quadruplex (**QI**) was prepared from (5′-(G4T4G3)2-3′), **QII** is QDNA derived from the 22-mer HTelo oligomer (5′-(AG3(T2AG3)3)-3′), and **QIII** is the G-quadruplex strand from the 22-mer human oncogene promoter c-myc (5′-(TGAG3TG3TAG3TG3TA2)-3′). Remarkably, incubation of **4** with **QIII** resulted in virtually complete quenching of NIR luminescence (Figure 4A). A significant reduction in the nanoaggregate particle size of **4** in the presence of **QIII** compared to other DNA structures investigated was additionally demonstrated in DLS studies (Figure 4B). A stronger interaction of **4** with **QIII** compared to dsDNA is also indicated by UV melting curve analysis (i.e., determination of the temperature (T_m_) at which 50% of DNA is denatured). The **QIII** oligomer exhibited a 6.5 °C increase in T_m_ in the presence of **4**, whereas the T_m_ of dsDNA only increased 2.4 °C (Figures S23–S24). Consistent with these results, luminescence binding assays indicated greater affinity of **4** for **QIII** DNA (K_d_ = 3.26 μM) compared to dsDNA (K_d_ = 11.7 μM) (Figures S25–S26). 

Since complete quenching of the NIR emission of **4** was achieved in the presence of **QIII** DNA, this DNA oligomer was selected as the digestion substrate in **4**/DNA ensembles for construction of label-free assays to monitor DNAse I activity. As a positive control and a proof of concept to test our design strategy, degradation of DNA by addition of Fenton’s reagent (1.4 mM FeSO_4_ + 36 mM H_2_O_2_) to a solution of the non-emissive **4**/**QIII** DNA ensemble resulted in the recovery of NIR luminescence (Figure S27) [53]. Thus, platinum complex **4** liberated upon DNA cleavage effectively self-assembles into emissive aggregates without interference from DNA fragmentation products.

**Figure 5:**
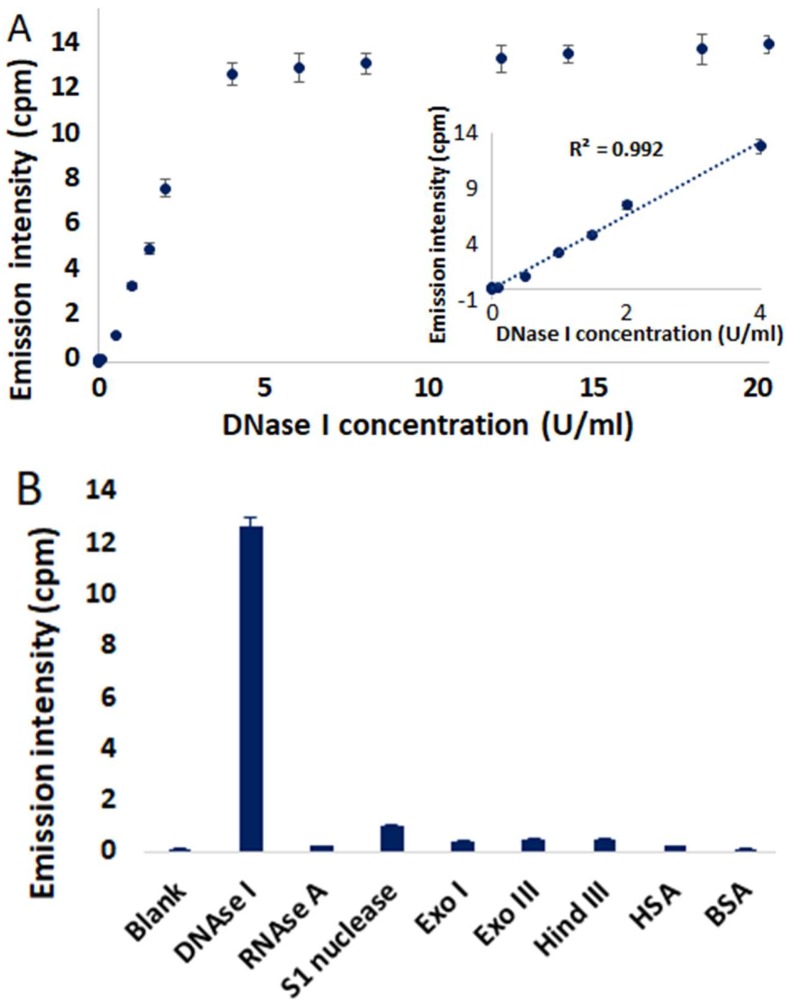
(**A**) Emission intensities of **4**/**QIII** DNA at 785 nm in the presence of different concentrations of DNAse I. Inset shows linear relationship with DNAse I concentration in the range of 0.01–4 U/mL. (**B**) Emission intensities of **4**/**QIII** DNA in the presence of different nucleases (4 U/mL) and proteins (8 µM). λ_ex_ = 445 nm. Error bars represent standard deviation (*n* = 3). All measurements were done after incubation at room temperature for 10 min.

The ability of **4**/**QIII** DNA ensembles to monitor DNAse I activity was next examined by measuring NIR emission in the presence of increasing concentrations of DNAse I (Figure 5A). Luminescence measurements were performed in 96 well plates using a solution of **4**/**QIII** DNA prepared from 4 μM **4** and 8 μM **QIII** DNA. The NIR emission intensity at 785 nm (indicative of DNA-free Pt complex aggregates) exhibited gradual enhancement in intensity as a function of DNAse I concentration and reached a plateau at ~6 U/mL DNAse I. Treatment of **4**/**QIII** DNA ensembles with heat-inactivated DNAse I failed to elicit a luminescence response, verifying that the catalytic activity of DNAse I is crucial for NIR emission (Figure S28). Since DNAse I is a Mg^2+^-dependent enzyme [9,12], the degradation of **4**/**QIII** DNA by DNAse I was performed in a reaction buffer without Mg^2+^, which also resulted in considerable attenuation of NIR emission (Figure S29). In the absence of **QIII**, addition of DNAse I to **4** in 9:1 Tris buffer:DMSO resulted in negligible change in its emission profile (Figure S30). These results confirm that NIR emission intensity of **4**/**QIII** DNA is correlated with **QIII** DNA cleavage by DNAse I.

The inset in Figure 5A reveals a linear relationship in the DNAse I concentration range of 0.01–4 U/mL. In addition, the detection limit of DNAse I is estimated to be 0.002 U/mL (3 × S_0_/S; S_0_ is the standard deviation and S is the slope of the calibration curve). Significantly, the **4**/**QIII** DNA ensemble is more sensitive in terms of detection of DNAse I activity than previously reported fluorescence-based DNAse I assays (Table S1). To address the selectivity of this method for DNAse I, other nucleases (RNAse A, S1 nuclease, Exonuclease I (Exo I), Exonuclease III (Exo III) and Hind III) and proteins (human serum albumin (HSA), bovine serum albumin (BSA)) were screened for their abilities to elicit NIR emission of **4**/**QIII** DNA. In each case minimal to no NIR emission was detected (Figure 5B), demonstrating the selectivity of this assay for DNAse I. Optimal assay pH was determined to be 7.5, and highest DNAse I activity was observed in the presence of 0.1 mM CaCl_2_ and 0.25 mM MgCl_2_ (Figures S31–S32).

**Figure 6:**
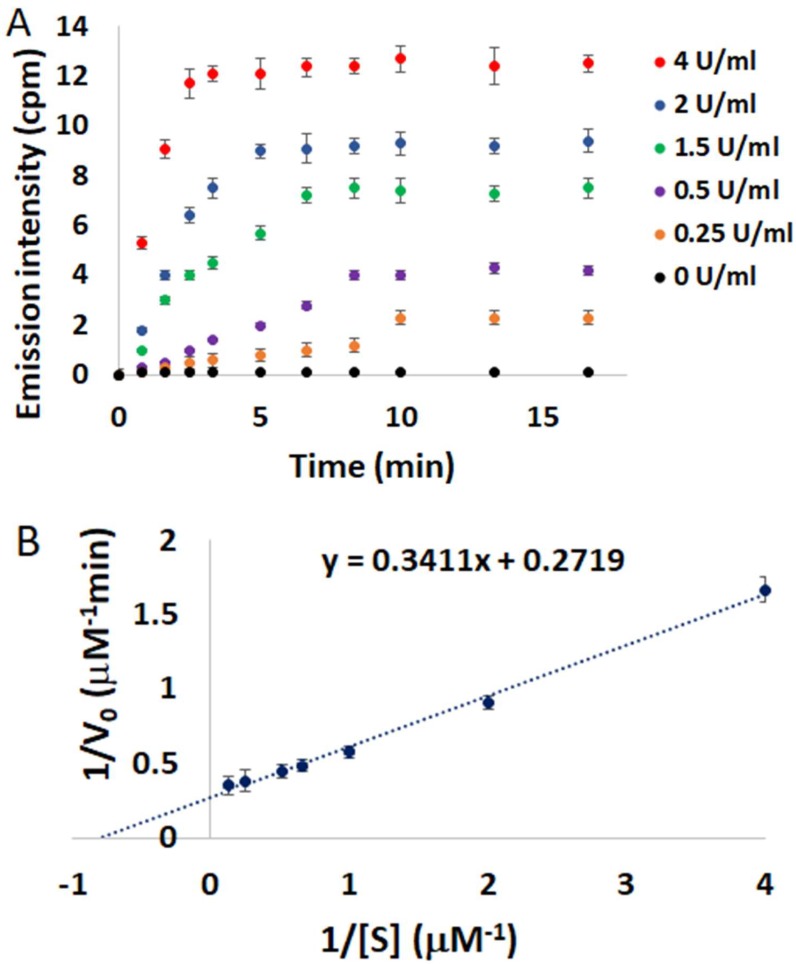
(**A**) Emission intensities of **4**/**QIII** DNA at 785 nm versus time at different DNAse I concentrations, λ_ex_ = 445 nm, [**4**] = 4 µM, [**QIII**] = 8 µM. (**B**) Lineweaver-Burk double reciprocal plot of initial digestion rate (1/*V_0_*) versus substrate concentration (1/[S]). Error bars represent standard deviation (*n* = 3).

Time curves for digestion of **4**/**QIII** DNA as a function of DNAse I concentration (0–4 U/mL) are displayed in Figure 6A. In the absence of DNAse I, negligible NIR emission can be detected over the incubation time. However, a rapid enhancement in the NIR emission signal is observed in the presence of 0.25 U/mL DNAse I. The emission signal plateaus after only 10 min, demonstrating the quick response of this assay to DNAse I activity. The digestion reaction rate increased gradually in the presence of higher concentrations of DNAse I (Figure 6A), and a linear relationship between initial digestion rate (*V_0_*) and DNAse I concentration was observed (Figure S33). In order to further verify the validity of this method to study DNAse I kinetics, the initial digestion rates (*V_0_*) were determined as a function of **4**/**QIII** DNA concentration ([S]). A Lineweaver-Burk double reciprocal plot of 1/*V_0_* versus 1/[S] revealed a linear correlation (Figure 6B) with a Michaelis–Menten constant (K_m_) of 1.26 ± 0.3 µM. This calculated K_m_ value is in good agreement with previously reported K_m_ values for DNAse I, which fall in the range of 0.4–2.19 µM [21,22]. These results show that the **4**/**QIII** DNA ensemble is an efficient real-time assay of DNAse I activity and its kinetic parameters.

**Figure 7:**
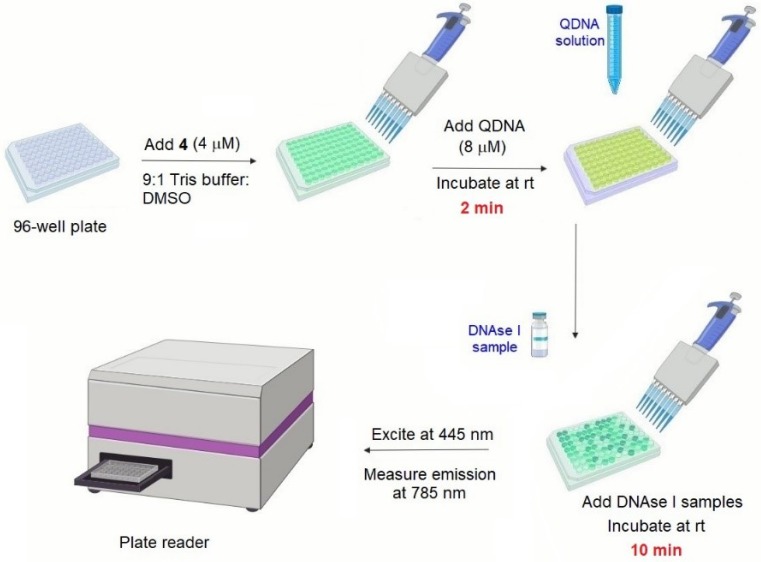
Schematic illustration of the general procedure of the developed DNAse I assay.

In order to evaluate the performance of **4**/**QIII** DNA as a DNAse I sensor in complex matrices, this method was used in detecting DNAse I activity in human serum samples. Various concentrations of DNAse I (0–4 U/mL) were added to human serum samples and subjected to the assay procedure. A linear correlation between NIR emission signal of **4** and DNAse I concentration in human serum samples was observed (Figure S34). In addition, assessing DNAse I activity in human serum samples spiked with 4 different concentrations of DNAse I (0.15, 0.5, 1 and 4 U/mL) revealed satisfactory reproducibility and precision (Table S2). These results demonstrate the potential of this system to detect DNAse I activity in real clinical samples. The assay was validated for high-throughput screening (HTS) mode by calculating the Z’ factor, representing the ratio of data signal variability (standard deviation) to dynamic range (i.e., difference in luminescence signal for positive and negative controls) [54]. The mean Z’ factor of the assay is 0.54 (see Supporting Information), which is indicative of a high quality assay (Z’ ≥ 0.5) [54]. This was accompanied by signal-to-background (S/B) ratio and signal-to-noise (S/N) ratio of 12.7 and >2000, respectively. These parameters confirm the potential suitability of **4**/**QIII** DNA assay for HTS of DNAse I activity. A schematic illustration of the general procedure of the reported assay featuring HTS compatibility is displayed in Figure 7.

**Figure 8:**
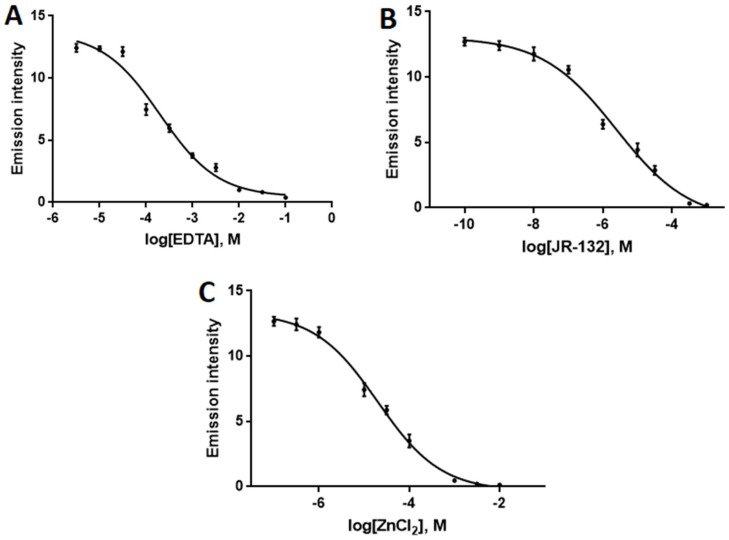
Dose-response curve in the presence of different concentrations of (**A**) EDTA, (**B**) JR-132, and (**C**) ZnCl_2_ based on NIR emission intensity of **4**/**QIII** ensemble. λ_ex_ = 445 nm, [**4**] = 4 μM, [**QIII**] = 8 μM, [DNAse I] = 4 U/mL. Error bars represent standard deviation (*n* = 3).

As a further test of this assay, the ability of **4**/**QIII** DNA ensembles to identify DNAse I inhibitors also was examined. Identification of DNAse I inhibitors is attracting increasing attention as inhibition of DNAse I may exert tissue-protective effects against necrosis and radiation injury [11,55,56]. In addition, DNAse I inhibitors are proposed to be effective in the treatment of male infertility through prevention of sperm DNA fragmentation [57]. The inhibitor assay was performed by monitoring the NIR emission intensity of **4**/**QIII** DNA in the presence of DNAse I and varying concentrations of three known DNAse I inhibitors: EDTA, JR-132 (1,4-phenylene-bis-aminoguanidine hydrochloride), and ZnCl_2_. Inhibitor IC_50_ values were determined from plots of log[inhibitor] vs. NIR emission intensity (Figure 8). Experimentally calculated IC_50_ values for the three inhibitors (EDTA: 202 μM, JR-132: 2.29 μM, ZnCl_2_: 20.7 μM) are all in excellent agreement with previously reported values (Table S3) [21]. Thus, the **4**/**QIII** DNA luminescence assay is effective for direct determination of DNAse I activity and detection/quantification of DNAse I inhibition.

## 3. Materials and Methods

A description of all materials and methods used in this study is provided in the Supporting Information.

### 3.1. UV–Visible Spectroscopy

UV–visible spectra were obtained using quartz cuvettes on a Varian Cary 100-Scan dual-beam spectrophotometer (Agilent, Santa Clara, CA, USA). Each measurement was done in duplicate and compared to solvent blank. Blank samples were prepared using HPLC grade solvents.

### 3.2. Fluorescence Spectroscopy

Fluorescence spectra were obtained at room temperature using an Agilent Cary Eclipse fluorescence spectrophotometer (Agilent, Santa Clara, CA, USA) in quartz cuvettes or white 96-well plates (Costar, Corning, NY, USA). Each measurement was done in triplicate. Sample stock solutions were prepared in HPLC grade DMSO. DNA stock solutions were prepared in Tris buffer (50 mM NaCl, 2 mM Tris, pH 7.5).

### 3.3. General Procedure for Multiwell DNAse I Assay

A reaction mixture (total volume of 100 µL) was prepared using 40 µL of stock solution of **QIII** (20 µM), 40 µL of probe **4** (10 µM), and 20 µL from the various concentrations tested of DNAse I. The probe **4** and **QIII** had final concentrations in the reaction mixture of 4 and 8 µM, respectively. Stock solutions were prepared using 9:1 Tris buffer (10 mM Tris-HCl, 0.25 mM MgCl_2_, 0.1 mM CaCl_2_, pH 7.5):DMSO. Tris buffer was prepared using nuclease-free water. The reaction mixtures were prepared in white 96-well plates (Costar, Corning, NY, USA) and incubated at room temperature for 2 min. Varying concentrations of DNAse I prepared in Tris buffer (10 mM Tris-HCl, 0.25 mM MgCl_2_, 0.1 mM CaCl_2_, pH 7.5) were added to the reaction mixture and incubated at room temperature for 10 min. Fluorescence spectra were obtained using Agilent Cary Eclipse fluorescence spectrophotometer (Agilent, Santa Clara, CA, USA), λ_ex_ = 445 nm. NIR emission intensity at 785 nm was detected.

### 3.4. Lineweaver-Burk Plot

The assay was performed in multiwell plates as described in the general procedure above using a reaction mixture (100 µL) prepared from a starting solution of **4**/**QIII** (4 and 8 μM, respectively, see General Procedure) diluted with buffer to obtain varying substrate concentrations of **QIII** (0, 0.25, 0.5, 1, 1.5, 2, 4 and 8 µM). DNAse I (4 U/mL) was added and mixtures were incubated at room temperature for 10 min. followed by fluorescence measurements. The initial digestion rates (*V_0_*) were measured from time curves of digestion reactions.

### 3.5. Detection of DNAse I in Human Serum

Human serum from human male AB plasma was purchased from Sigma-Aldrich (St. Louis, MO, USA). DNAse I assay was performed as previously described. Different concentrations of DNAse I were prepared in human serum and added to reaction mixtures.

### 3.6. Determination of IC_50_ Values of DNAse I Inhibitors

DNAse I assay was performed as previously described in the presence of DNAse I (4 U) and various concentrations of the inhibitor. Stock solutions of the inhibitors were prepared in Tris buffer (10 mM Tris-HCl, pH 7.5). The IC_50_ values were calculated by plotting log[inhibitor] versus NIR emission intensity of **4**. The dose-response curves were analyzed by nonlinear regression using GraphPad Prism 8.0 (GraphPad Software, Inc., La Jolla, CA, USA).

## 4. Conclusions

In summary, we have developed a new G quadruplex-based luminescence assay for sensitive, label-free, rapid and real-time detection of DNAse I activity and inhibition. The developed assay is the most sensitive of any luminescence-based assay available for DNAse I activity and has further advantages of HTS compatibility and cost efficiency. A structurally novel and neutral diplatinum(II) complex (**4**) has been easily prepared via cyclometalation of a bis(pyridyl)-bis(benzothiazole) tetraarylethylene derivative. The complex exhibits switchable near-IR luminescence at 785 nm as a function of solvent-induced aggregation. Association of **4** with DNA was found to result in varying degrees of NIR emission quenching as a function of DNA structure, and complete luminescence quenching was observed in the presence of G-quadruplex DNA derived from the human c-myc oncogene. Subsequent DNA degradation liberates **4** which then self-assembles to produce a turn-on luminescence signal. The switchable NIR emission of this **4**/DNA ensemble was successfully used to develop a fast (10 min), sensitive (LOD = 0.002 U/mL), and label-free assay for the endonuclease DNAse I that possesses distinct advantages over all previously reported DNAse I assays. Furthermore, the assay can be easily modified to allow screening for DNAse I inhibitors. Assay experiments were performed in multiwell plates and are compatible with high throughput screening techniques. Significantly, the suitability of this method for clinical use is demonstrated by utilizing **4**/**QIII** DNA system for sensitive detection of DNAse I in human serum samples.

Finally, it is notable that the variable response of **4** based on DNA structure should enable further optimization of the system for different applications in biosensor technology. Moreover, the organometallic tetra-arylethylene scaffold utilized in this study is representative of a versatile molecular framework well-suited for development of additional organic and metal-organic bioprobes for use in diverse chemical biology applications. In particular, we envision that structural modification of the diplatinum complexes described here may ultimately facilitate use of these agents for in vivo monitoring of cellular events as activatable NIR luminescent probes.

## Supplementary Materials

The following are available online. Detailed synthetic procedures, UV–visible and emission spectra for **2**–**4**, spectroscopic data for **4**, DNA structures, DLS data, copies of ^1^H and ^13^C NMR spectra for all new compounds.
