# Supplementary material for: Expanding the Toolbox for Label-Free Enzyme Assays: A Dinuclear Platinum(II) Complex/DNA Ensemble with Switchable Near-IR Emission
Source: Molecules. 2019 Dec 1;24(23):4390. doi: 10.3390/molecules24234390 (PMC6930566; doi:10.3390/molecules24234390)
Supplement: Supplementary file 1 [file molecules-24-04390-s001.pdf]

Supporting Information

Expanding the toolbox for label-free enzyme assays: A dinuclear platinum(II) complex/DNA ensemble with switchable near-IR emission

Moustafa T. Gabr and F. Christopher Pigge

Department of Chemistry, University of Iowa, Iowa City, Iowa 52242, United States

chris-pigge@uiowa.edu

Contents

Experimental	S3
UV-vis absorption spectrum of **B**	S8
UV-vis absorption spectrum of **2**	S8
UV-vis absorption spectrum of **E**	S9
UV-vis absorption spectrum of **3**	S9
UV-vis absorption spectrum of **G**	S10
UV-vis absorption spectrum of **4**	S10
Emission profile of **2** in DMSO and 9:1 Tris buffer	S11
Emission profile of **3** in DMSO and 9:1 Tris buffer	S11
DLS particle size analysis of **4** in 9:1 Tris buffer:DMSO	S12
^{1}H NMR spectra of **4** in DMSO-d_6 and 9:1 D_2O:DMSO-d_6	S12
The UV-vis absorbance values of **4** in 9:1 Tris buffer:DMSO at different concentrations of **4**	S13
Emission profile of **4** in 9:1 Tris buffer:DMSO at different temperatures	S13
Sequences of DNA structures used in this study	S14
Emission profile of **4** in the absence and the presence of ssDNA	S14
Emission profile of **4** in the absence and the presence of dsDNA	S15
Emission profile of **4** in the absence and the presence of **QI**, **QII** and **QIII**	S15
DLS particle size analysis of **4** in the presence of ssDNA	S16
DLS particle size analysis of **4** in the presence of dsDNA	S16
DLS particle size analysis of **4** in the presence of **QI**	S17
DLS particle size analysis of **4** in the presence of **QII**	S17
DLS particle size analysis of **4** in the presence of **QIII**	S18
Changes in UV-vis absorption at 260 nm of dsDNA with and without **4**	S18

Changes in UV-vis absorption at 295 nm of **QIII** with and without **4** S19

Saturation binding isotherm using various concentrations of dsDNA towards **4** S19

Saturation binding isotherm using various concentrations of **QIII** towards **4** S20

Emission profile of **4** and **4**/**QIII** in the absence and the presence of Fenton's reagent S20

Emission profile of **4**/**QIII** ensemble in the presence of DNAse I and heat denatured DNAse I S21

Emission profile of **4**/**QIII** ensemble in the presence of DNAse I with and without $MgCl_2$ S21

Emission profile of **4** in the presence and absence of DNAse I S22

NIR Emission intensity of **4/QIII** ensemble in the presence of DNAse I at different pH values S22

NIR Emission intensity of **4/QIII** ensemble in the presence of DNAse I at different combinations of $MgCl_2$ and $CaCl_2$ S23

Initial cleavage velocity (V_0) of **4**/**QIII** ensemble as a function of DNAse I concentration S23

The linear correlation of NIR emission intensity of **4**/**QIII** ensemble and DNAse I concentrations in human serum samples S24

Comparison of the developed DNAse I assay to other fluorescence-based DNAse I assays S25

Determination of DNAse I activity in diluted human serum S26

Determination of IC_{50} values of known DNAse I inhibitors S27

References S28

NMR spectra of all compounds S29

Mass spectra of all compounds S46

Experimental:

General

All commercially available starting materials, reagents, and solvents were used as supplied unless otherwise stated. Potassium tetrachloroplatinate was purchased from Pressure Chemical Co. Palladium (II) acetate was purchased from Sigma Aldrich. 2-Aminothiophenol was purchased from TCI chemicals. 3-Formylphenylboronic acid was purchased from Combi-Blocks. Reported yields are isolated yields. Proton (^{1}H) and carbon (^{13}C) NMR were collected on a Bruker NMR spectrometer at 300 MHz or 400 MHz for ^{1}H and 75 MHz or 100 MHz for ^{13}C. Chemical shifts (δ) are reported in parts-per million (ppm) relative to residual undeuterated solvent. ^{195}Pt NMR was collected on a Bruker NMR spectrometer at 86 MHz and referenced externally against potassium tetrachloroplatinate(II) in D_2O. Melting points were recorded using a capillary melting point apparatus and are uncorrected. High resolution mass spectra were obtained in positive ion mode using electrospray ionization (ESI) on a double-focusing magnetic sector mass spectrometer or electron ionization time of flight mass spectroscopy (EI-TOF MS). Elemental analyses were performed using CE-440 Elemental Analyzer-Exeter Analytical, Inc. Particle size distribution analysis was measured by dynamic light scattering (DLS) using a Malvern Zetasizer Nano ZS instrument. Oligonucleotides were obtained from Integrated DNA Technologies (Coralville, IA) and purified by HPLC using a C_{18} reverse-phase column (Varian, Inc.). The HPLC purity of oligonucleotides used in this study was above 95%. The G-quadruplexes from **QI**, **QII** and **QIII** were formed as previously described by heating the corresponding oligonucleotide solution (1 mM) in potassium phosphate buffer at 95 °C for 15 min and allowing the solution to equilibrate at room temperature overnight.[1] The synthesis and photophysical properties of platinum(II) complex **1** were previously reported.[2]

Synthetic procedures:

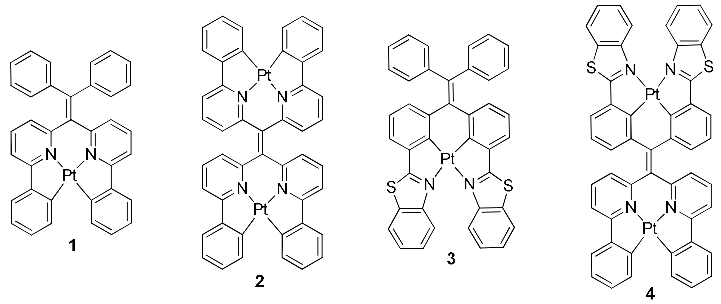

Figure S1. Chemical structures of platinum(II) complexes **1-4**.

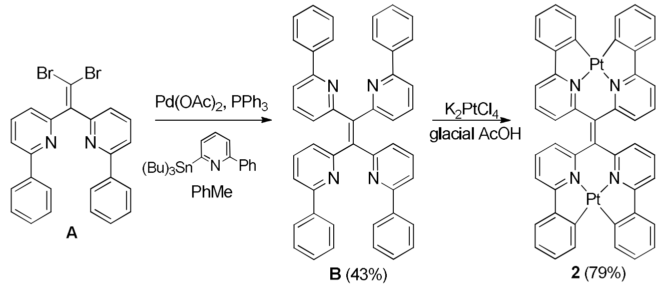

Scheme S1. Synthesis of platinum(II) complex **2**.

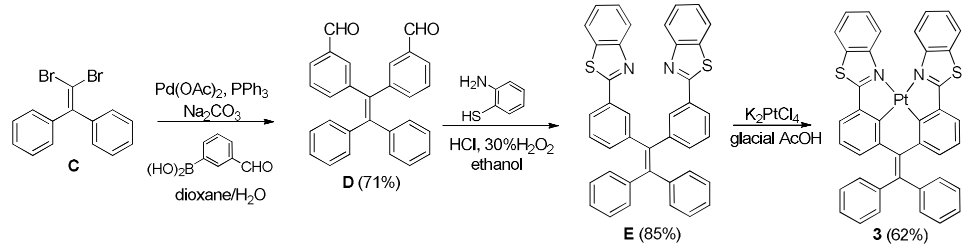

Scheme S2. Synthesis of platinum(II) complex **3**.

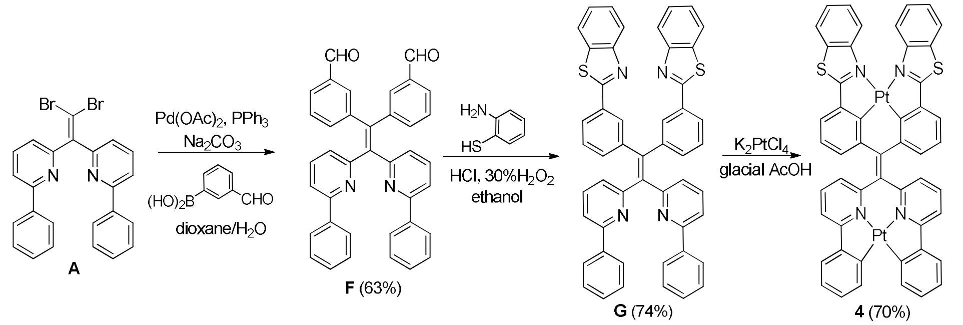

Scheme S3. Synthesis of platinum(II) complex **4**.

1,1,2,2-Tetrakis(6-phenylpyridin-2-yl)ethene (B)

Compound **A**[2] (377 mg, 0.76 mmol) was dissolved in PhMe (25 mL). The flask was charged with $Pd(OAc)_2$ (108 mg, 0.48 mmol), PPh_3 (524 mg, 2.00 mmol) and 2-phenyl-6-(tributylstannyl)pyridine[3] (1.37 g, 3.10 mmol). The reaction was heated to reflux under argon overnight. The organic solvent was evaporated under reduced pressure and the crude product was dissolved in MeCN (50 mL) and filtered over Celite. The MeCN layer was extracted with hexane (2 × 25 mL), then the hexane layer was discarded. The MeCN layer was concentrated under reduced pressure and the crude product was purified by flash column chromatography using 40% ethyl acetate in hexane as eluent to yield **B** (212 mg, 43%) as a yellowish white solid. Mp 129-132°C. ^{1}H NMR (400 MHz, C*D*Cl$_3$) δ 7.56-7.66 (m, 16H), 7.90 (d, 4H, *J* = 7.8 Hz), 8.02 (t, 4H, *J* = 7.8, 7.8 Hz), 8.28-8.33 (m, 8H). ^{13}C NMR (75 MHz, C*D*Cl$_3$) δ 119.4, 120.2, 126.9, 128.6, 128.9, 136.7, 137.5, 139.3, 155.8, 156.2. HRMS (ESI): calcd for $C_{46}H_{33}N_4$ $[M+H]^+$, 641.2705; found, 641.2709.

Complex 2

Compound **B** (22.0 mg, 0.034 mmol) and K_2PtCl_4 (29.0 mg, 0.068 mmol) were mixed in glacial acetic acid (3 mL), and the resulting mixture was heated under reflux overnight. After cooling to rt, the crude reaction mixture was poured into H_2O (30 mL) and the resulting yellow precipitate was collected by vacuum filtration. The crude material was purified by flash column chromatography using 100% dichloromethane to yield **2** (27.5 mg, 79%) as a yellow solid. Mp >220°C. ^{1}H NMR (400 MHz, DMSO-*d*$_6$) δ 7.01-7.05 (m, 4H), 7.29-7.41 (m, 12 H), 7.85 (d, 4H, *J* = 7.6 Hz), 7.91-7.95 (m, 4H), 8.52 (d, 4H, *J* = 7.9 Hz). ^{13}C NMR (100 MHz, methanol-*d*$_4$) δ 120.1, 122.3, 123.4, 127.2, 127.6, 127.8, 129.5, 129.8, 138.6, 140.5, 149.8, 158.6. MS (EI): calcd for $C_{46}H_{28}N_4Pt_2$ $[M]^{+,}$ 1026.1; found, 1026.1.

3,3'-(2,2-Diphenylethene-1,1-diyl)dibenzaldehyde (D)

Compound **C**[4] (338 mg, 1.00 mmol) was dissolved in 50 mL of dioxane : water (4 : 1). The flask was charged with Na_2CO_3 (690 mg, 5.00 mmol), $Pd(OAc)_2$ (54 mg, 0.24 mmol), PPh_3 (262 mg, 1.00 mmol) and 3-formylphenylboronic acid (750 mg, 5.00 mmol). The reaction was heated to reflux under argon overnight. After cooling, the reaction mixture was diluted with water and extracted with ethyl acetate (2 × 50 mL)

and the combined organic fractions were dried over anhydrous Na_2SO_4, filtered, and concentrated under reduced pressure. The crude product was purified by flash column chromatography using 5% ethyl acetate in hexane as eluent to yield **D** (275 mg, 71%) as a white solid. Mp 115-116°C. ^{1}H NMR (400 MHz, C*D*Cl$_3$) δ 7.03-7.06 (m, 4H), 7.10-7.13 (m, 6H), 7.26-7.34 (m, 4H), 7.54-7.56 (m, 2H), 7.64-7.66 (m, 2H), 9.80 (s, 2H). ^{13}C NMR (100 MHz, C*D*Cl$_3$) δ 128.3, 129.0, 129.1, 129.8, 132.2, 133.7, 137.3, 138.3, 139.0, 143.5, 144.9, 145.1, 193.1. HRMS (ESI): calcd for $C_{28}H_{21}O_2$ $[M+H]^+$, 389.1542; found, 389.1539.

2,2'-((2,2-Diphenylethene-1,1-diyl)bis(3,1-phenylene))bis(benzothiazole) (E)

Compound **D** (415 mg, 1.07 mmol) and 2-aminothiophenol (266 mg, 2.13 mmol) were dissolved in ethanol (12 mL). After addition of 37.2% aq. HCl (0.52 mL, 6.20 mmol) and 30% aq. H_2O_2 (1.33 mL, 13.00 mmol), the reaction mixture was stirred at room temperature for 2 h. The reaction mixture was diluted with water (100 mL) and extracted with ethyl acetate (2 × 50 mL) and the combined organic fractions were dried over anhydrous Na_2SO_4, filtered, and concentrated under reduced pressure. The crude product was purified by flash column chromatography using 3% ethyl acetate in hexane as eluent to yield **E** (544 mg, 85%) as a yellowish white solid. Mp 160-162°C. ^{1}H NMR (400 MHz, C*D*Cl$_3$) δ 7.23-7.27 (m, 10H), 7.33-7.38 (m, 4H), 7.44-7.46 (m, 2H), 7.53-7.55 (m, 2H), 7.93-7.95 (m, 4H), 8.01 (dt, J = 7.5, 1.4 Hz, 2H), 8.13 (d, J= 8.1 Hz, 2H). ^{13}C NMR (100 MHz, C*D*Cl$_3$) δ 122.6, 124.2, 126.1, 126.9, 127.3, 128.1, 129.0, 129.7, 131.7, 132.3, 134.3, 135.1, 136.1, 139.9, 144.0, 144.2, 145.0, 155.1, 169.0. HRMS (ESI): calcd for $C_{40}H_{27}N_2S_2$ $[M+H]^+$, 599.1616; found, 599.1614.

Complex 3

Using the procedure given for the preparation of **2**, compound **E** (150 mg, 0.25 mmol) and K_2PtCl_4 (104 mg, 0.25 mmol) gave **3** (123 mg, 62%) as a yellow solid after purification by flash column chromatography using 100% dichloromethane as eluent. Mp >230°C. ^{1}H NMR (400 MHz, DMSO-d_6) δ 7.09-7.14 (m, 6H), 7.17-7.22 (m, 4H), 7.25-7.28 (m, 2H), 7.38-7.45 (m, 4H), 7.49-7.53 (m, 2H), 7.78-7.80 (m, 2H), 7.98-8.01 (m, 2H), 8.08-8.11 (m, 2H). ^{13}C NMR (75 MHz, DMSO-d_6) δ 125.3, 126.0, 127.7, 128.1, 128.7, 129.2, 129.7, 131.5, 132.0, 132.2, 132.4, 135.8, 136.2, 137.4, 140.4, 141.7, 146.6, 156.5, 169.9. HRMS (EI): calcd for $C_{40}H_{24}N_2PtS_2$ $[M]^+$, 791.1029; found, 791.1035.

3,3'-(2,2-Bis(6-phenylpyridin-2-yl)ethene-1,1-diyl)dibenzaldehyde (F)

Using the procedure given for the preparation of **D**, coupling of **A**[2] (1.51 g, 3.07 mmol) and 3-formylphenylboronic acid (2.30 g, 15.34 mmol) gave **F** (1.05 g, 63%) as a light yellow solid after purification by flash column chromatography using 10% ethyl acetate in hexane followed by 20% ethyl acetate in hexane as eluent. Mp 137-139°C. ^{1}H NMR (300 MHz, C*D*Cl$_3$) δ 7.25-7.27 (m, 2H), 7.46-7.49 (m, 6H), 7.51-7.55 (m, 2H), 7.63-7.69 (m, 8H), 7.74-7.78 (m, 2H), 7.88-7.92 (m, 4H), 10.03 (s, 2H). ^{13}C NMR (75 MHz, C*D*Cl$_3$) δ 118.6, 124.5, 126.6, 126.8, 127.9, 128.2, 128.6, 128.7, 132.2, 136.2, 136.5, 138.8, 142.3, 142.6, 143.8, 156.4, 159.1, 191.7. HRMS (ESI): calcd for $C_{38}H_{27}N_2O_2$ $[M+H]^+$, 543.2073; found, 543.2072.

2,2'-((2,2-Bis(6-phenylpyridin-2-yl)ethene-1,1-diyl)bis(3,1-phenylene))bis(benzothiazole) (G)

Using the procedure given for the preparation of **E**, compound **F** (364 mg, 0.67 mmol) and 2-aminothiophenol (169 mg, 1.35 mmol) gave **G** (373 mg, 74%) as a yellow solid after purification by flash column chromatography using 10% ethyl acetate in hexane as eluent. Mp 171-172°C. ^{1}H NMR (300 MHz, C*D*Cl$_3$) δ 7.28 (d, J = 7.5 Hz, 2H), 7.38-7.43 (m, 8H), 7.46-7.52 (m, 6H), 7.55-7.57 (m, 2H), 7.64-7.67 (m, 2H), 7.69-7.74 (m, 4H), 7.86 (d, J = 7.5 Hz, 2H), 8.10-8.14 (m, 6H). ^{13}C NMR (75 MHz, C*D*Cl$_3$) δ 118.1, 120.9, 122.5, 124.1, 124.5, 125.5, 125.6, 126.4, 127.8, 128.1, 128.2, 129.8, 132.8, 132.9, 134.4, 136.0, 138.8,

141.6, 143.0, 143.2, 153.4, 156.1, 159.2, 167.1. HRMS (ESI): calcd for $C_{50}H_{33}N_4S_2$ $[M+H]^+$, 753.2146; found, 753.2145.

Complex 4

Using the procedure given for the preparation of **2**, compound **G** (60.2 mg, 0.08 mmol) and K_2PtCl_4 (99.6 mg, 0.24 mmol) gave **4** (63.7 mg, 70%) as a yellow solid after purification by flash column chromatography using 25% ethyl acetate in hexane as eluent. Mp >230°C. ^{1}H NMR (300 MHz, $CDCl_3$) δ 7.29-7.34 (m, 6H), 7.41 (d, *J* = 7.4 Hz, 2H), 7.48-7.51 (m, 4H), 7.55-7.61 (m, 4H), 7.73 (d, *J* = 7.9 Hz, 2H), 7.82-7.84 (m, 2H), 7.91-7.93 (m, 2H), 8.00 (d, *J* = 7.6 Hz, 2H), 8.08 (d, *J* = 7.6 Hz, 2H), 8.31-8.33 (m, 2H). ^{13}C NMR (100 MHz, $CDCl_3$) δ 122.4, 124.0, 124.8, 126.0, 126.2, 127.2, 128.0, 128.8, 128.9, 129.0, 129.2, 129.7, 130.0, 130.3, 131.6, 132.5, 132.7, 134.2, 135.7, 139.2, 140.4, 142.6, 148.4, 154.6, 160.2, 167.7. ^{195}Pt NMR (86 MHz, $CDCl_3$) δ -2474.3, -2437.3. MS (EI): calcd for $C_{50}H_{28}N_4Pt_2S_2$ $[M]^{+,}$ 1138.1; found, 1138.1. Anal. Calcd $C_{50}H_{28}N_4Pt_2S_2$ (1138.11): C, 52.72; H, 2.48; N, 4.92. Found: C, 52.48; H, 2.41; N, 4.99.

Saturation binding isotherm

The interaction between **4** and DNA was quantified by measuring the change in NIR emission intensity of **4** (4 µM) in the presence of various concentrations of DNA (0-18 µM).
The experiment was performed in triplicate with results given as the mean ± SD. The fraction of **4** bound to DNA was plotted versus DNA concentration to yield binding isotherms. The fraction of **4** bound to DNA at each point of the titration was calculated following the changes of emission intensity at 785 nm, using the following equation:[5]

Fraction of **4** bound to DNA = $(I_{785}- I_{free})/(I_{bound}-I_{free})$

Where I_{785}: NIR emission intensity at 785 nm at the different molar ratios (**4**/DNA) investigated.

I_{free} :NIR emission intensity at 785 nm of **4** in the absence of DNA.

I_{bound} :NIR emission intensity at 785 nm of saturated sample of **4** with DNA.

The corresponding binding isotherms were analysed by GraphPad Prism 8.0 (GraphPad Software, Inc., La Jolla, CA, USA) by non-linear regression using a one site binding equation. The dissociation constants (K_d) values were calculated using the following equation:[6]

$$Y = Bmax*X/(Kd + X)$$

Where, X is the concentration of **4**, Y is change in emission intensity, Bmax is the maximum specific binding.

Validation of the assay for high-throughput screening (HTS):

The suitability of the assay for HTS was validated by calculating three screen parameters (signal-to-noise ratio (S/N), signal-to-background ratio (S/B) and *Z'* factor) using the following equations:[7]

$$S/N = \frac{mean\ signal\ of +ve\ control - mean\ background}{standard\ deviation\ of\ background}$$

$$S/B = \frac{mean\ signal\ of +v\ \ control}{mean\ background}$$

$$Z'\ factor = 1 - \frac{(S.D.+ve)+(S.D.-ve)}{(mean+ve)-(mean-ve)}$$

Where, S.D. +ve : Standard deviation of the positive control (i.e. in the presence of DNAse I)

S.D. –ve: Standard deviation of the negative control (i.e. in the absence of DNAse I)

Mean +ve : mean of the positive controls

Mean –ve : mean of the negative controls

Z′ factor was calculated using 10 plates run in 96-well format. Each plate contained 40 positive controls (DNAse I was added) and 40 negative controls (DNAse I was not added).

UV-Melting Experiments:

DNA melting temperature studies were carried out using quartz cells on an Agilent Cary Eclipse spectrophotometer equipped with a thermo-programmer. Melting curves were monitored at 260 nm for dsDNA and 295 nm for QDNA with a heating rate of 2 °C/min in the range of 24-98 °C. Melting temperatures were obtained by plotting the temperature versus change in absorbance. The point of inflection of the heating curve was calculated using the first derivatives from which the melting temperature was obtained. Experiments were performed in triplicate.

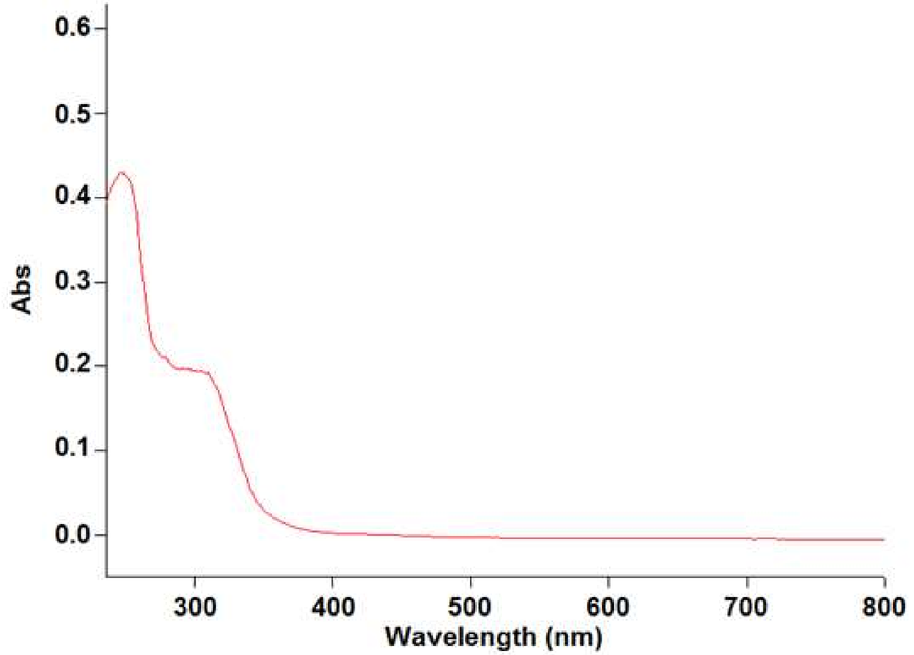

Figure S2. UV-vis absorption spectrum of **B** (DMSO, 2 μM).

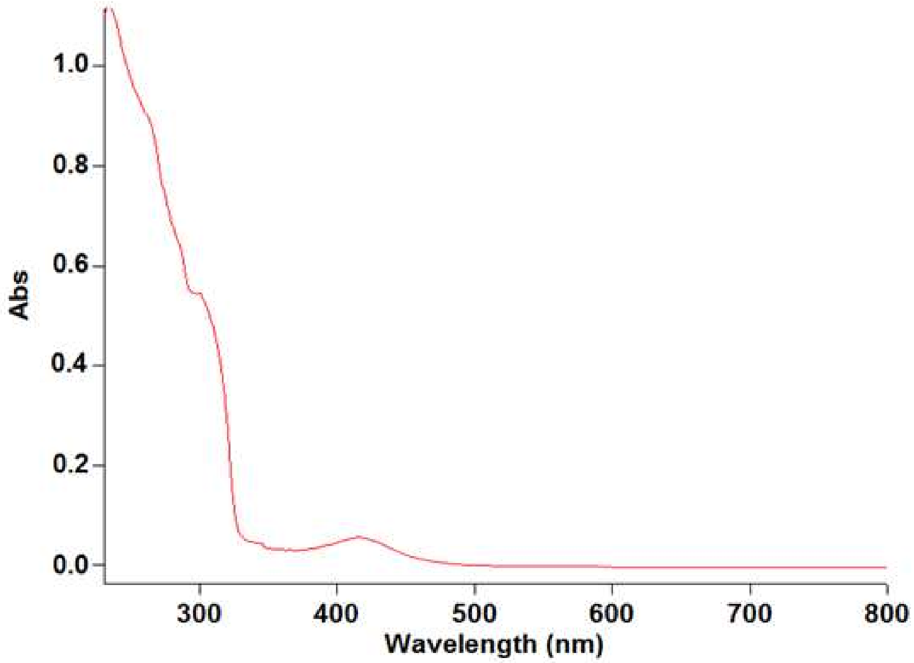

Figure S3. UV-vis absorption spectrum of **2** (DMSO, 6 μM).

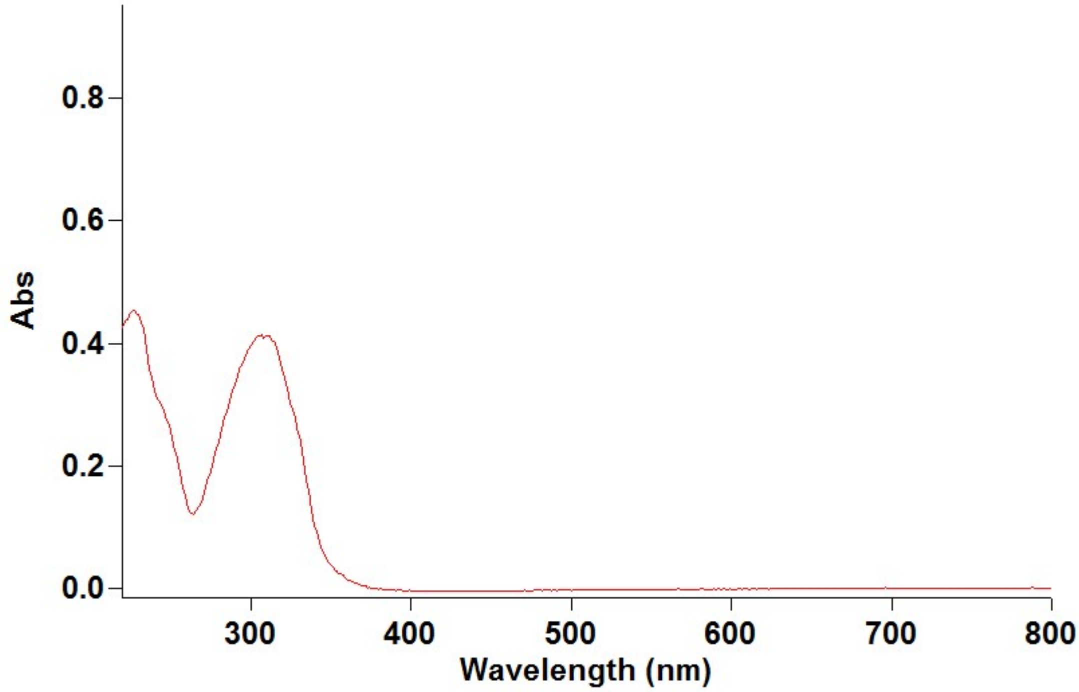

Figure S4. UV-vis absorption spectrum of **E** (DMSO, 4 μM).

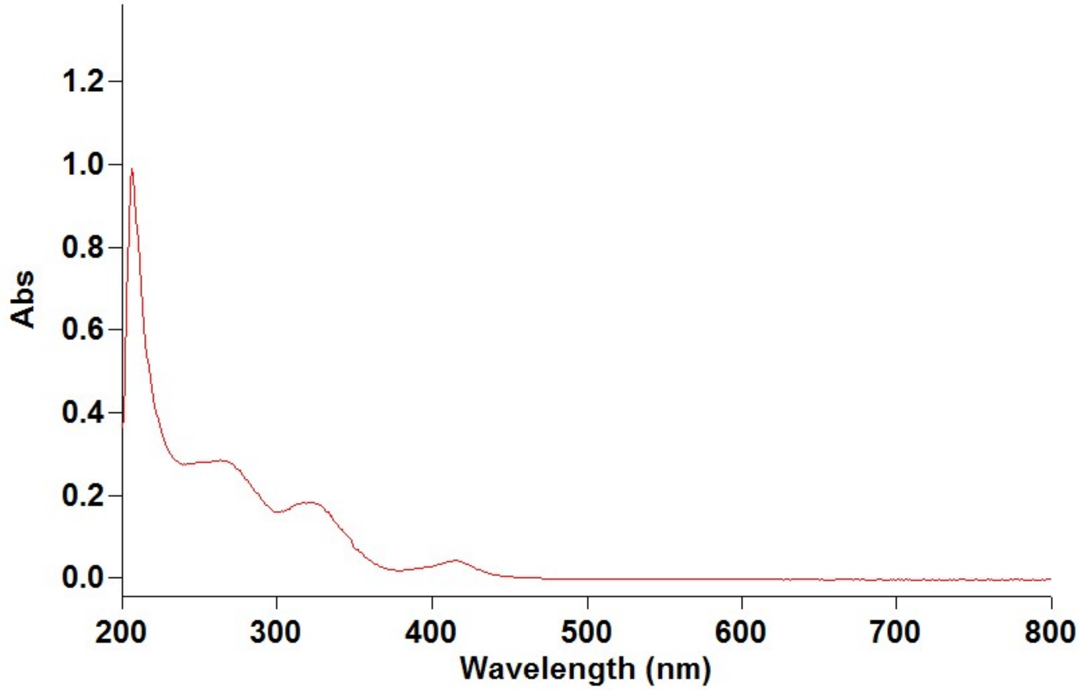

Figure S5. UV-vis absorption spectrum of **3** (DMSO, 4 μM).

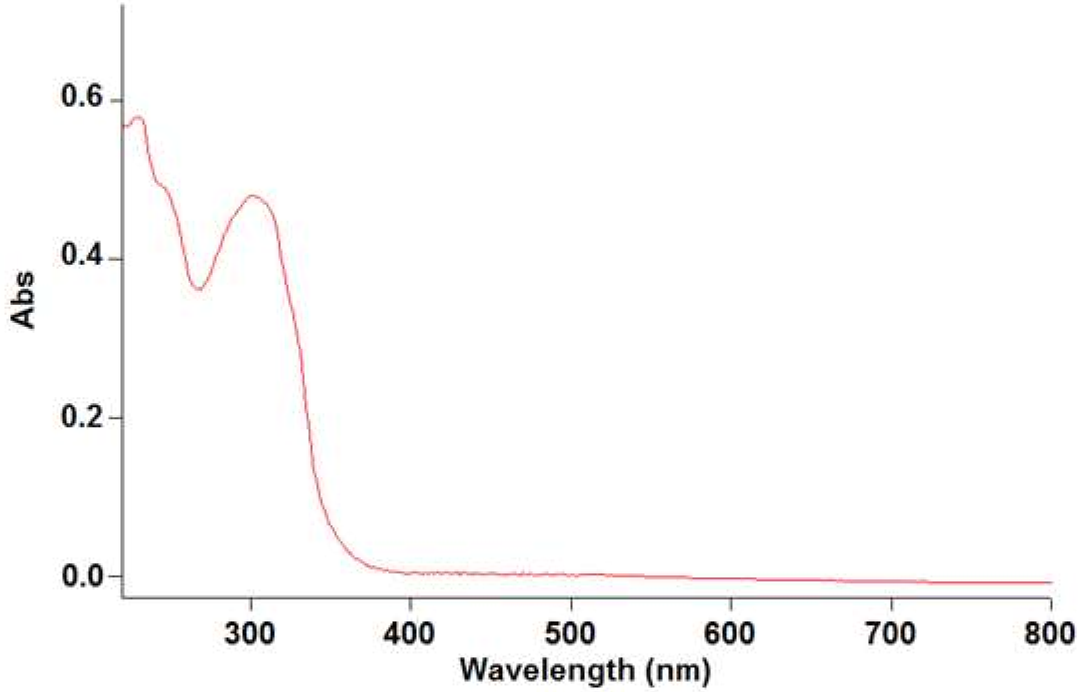

Figure S6. UV-vis absorption spectrum of **G** (DMSO, 4 μM).

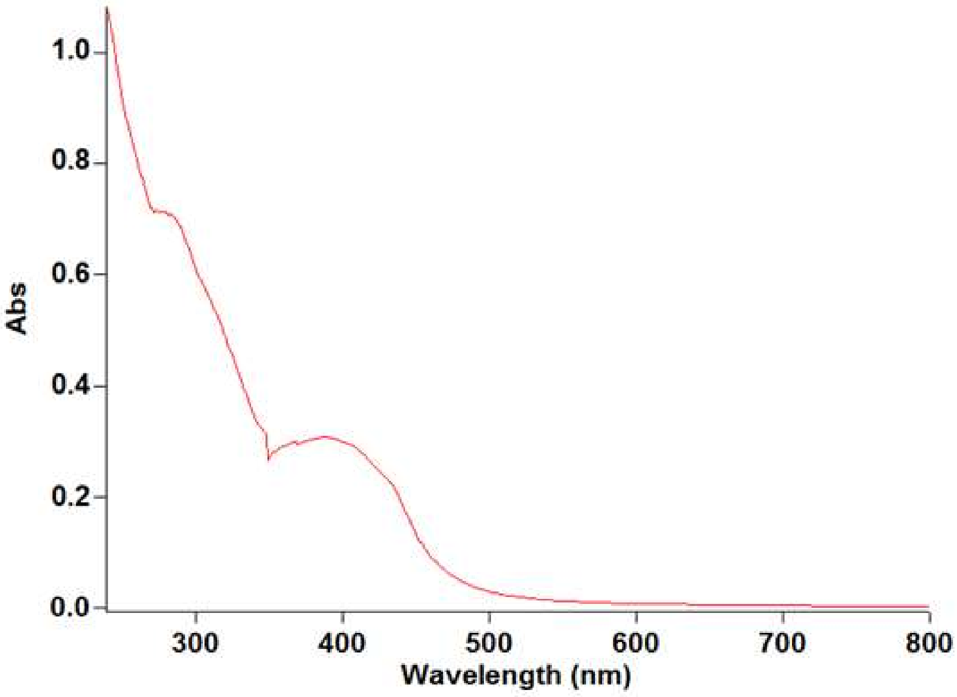

Figure S7. UV-vis absorption spectrum of **4** (DMSO, 4 μM).

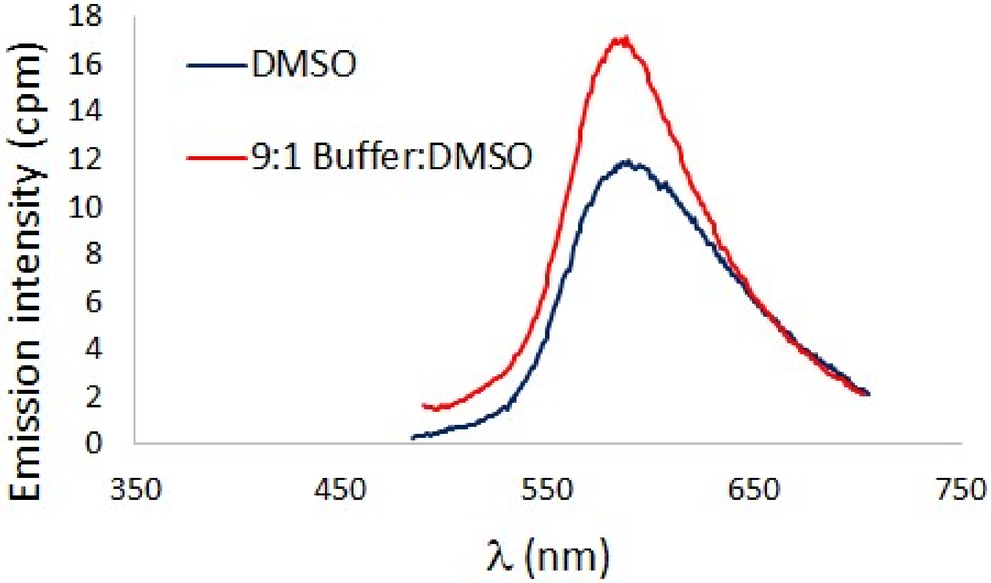

Figure S8. Emission profile of **2** in DMSO and 9:1 Tris buffer (10 mM Tris-HCl, pH 7.5):DMSO. λ_{ex} = 416 nm, [**2**] = 4 μM.

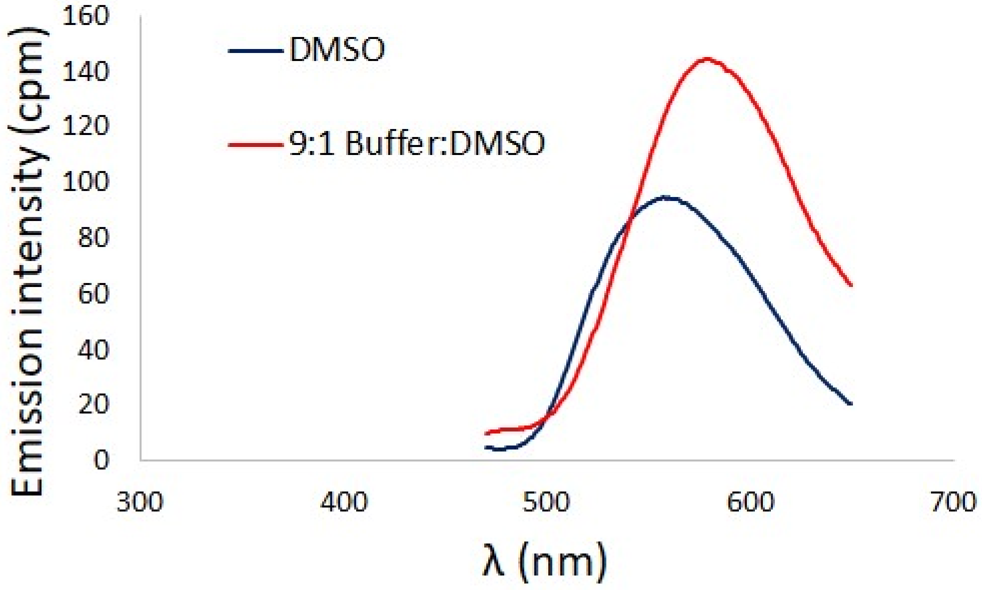

Figure S9. Emission profile of **3** in DMSO and 9:1 Tris buffer (10 mM Tris-HCl, pH 7.5):DMSO. λ_{ex} = 414 nm, [**3**] = 4 μM.

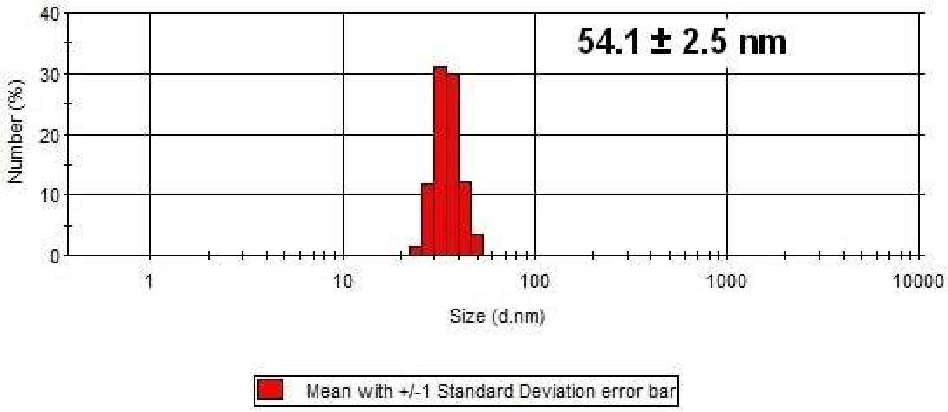

Figure S10. DLS particle size analysis of **4** (4 μM) in 9:1 Tris buffer (10 mM Tris-HCl, pH 7.5):DMSO.

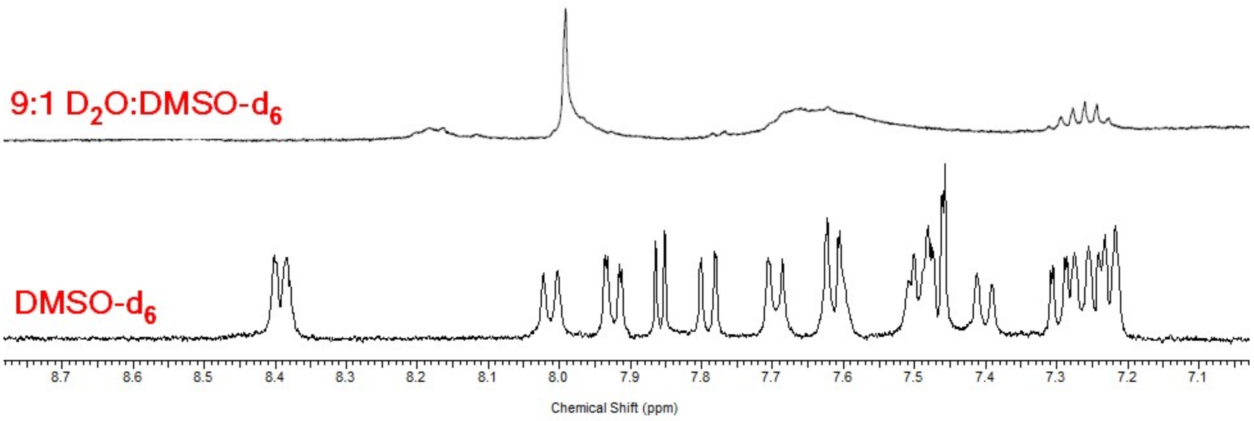

Figure S11. ^{1}H NMR spectra of **4** in DMSO-d_6 (bottom) and in 9:1 D_2O:DMSO-d_6 (top) collected on a Bruker NMR spectrometer at 400 MHz, [**4**] = 50 μM.

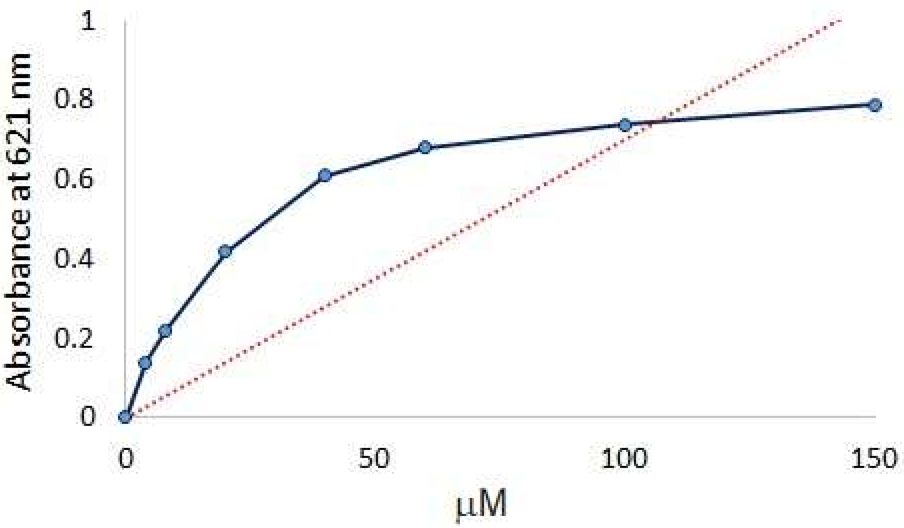

Figure S12. The UV-vis absorbance values of **4** at 621 nm in 9:1 Tris buffer (10 mM Tris-HCl, pH 7.5):DMSO at different concentrations of **4** (0-150 μM). Red tracer represents fitting of the data using straight line equation.

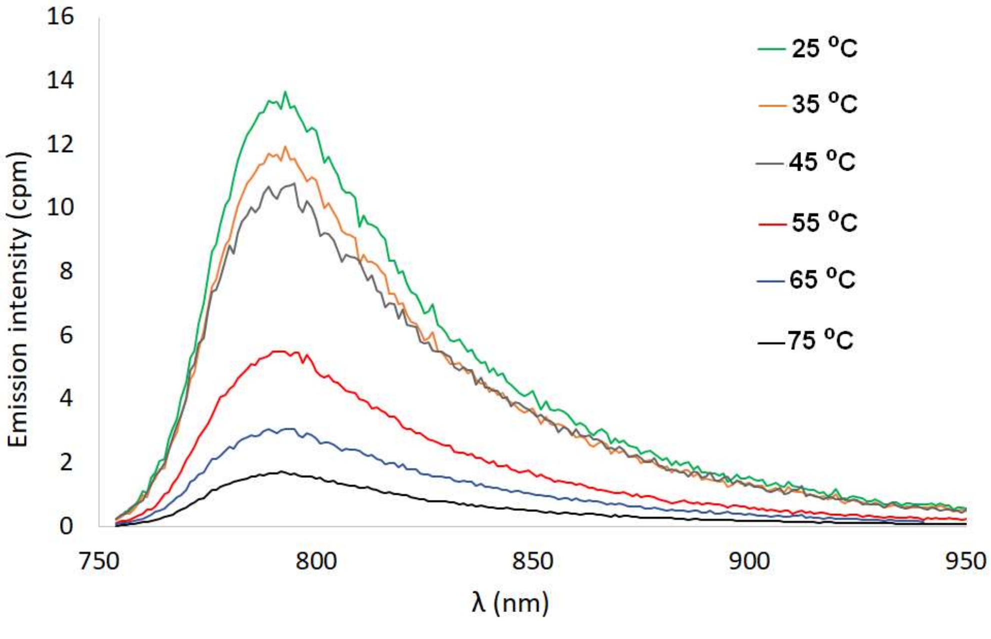

Figure S13. Emission profile of **4** in 9:1 Tris buffer (10 mM Tris-HCl, pH 7.5):DMSO at different temperatures. λ_{ex} = 445 nm, [**4**] = 4 μM.

**single-stranded DNA (ssDNA)**	5’ AAAAAAAAAAAAAAAAAAAAAA 3’
**double-stranded DNA (dsDNA)**	5’ CGCGAATTCGC 3’
	3’ GCGCTTAAGCG 5’
**G-quadruplex DNA (QDNA), QI**	5’ (GGGGTTTTGGG)$_2$ 3’
**QDNA, QII**	5’ AGGGTTAGGGTTAGGGTTAGGG 3’
**QDNA, QIII**	5’ TGAGGGTGGGTAGGGTGGGTAA 3’

Figure S14. Sequences of DNA structures used in this study.

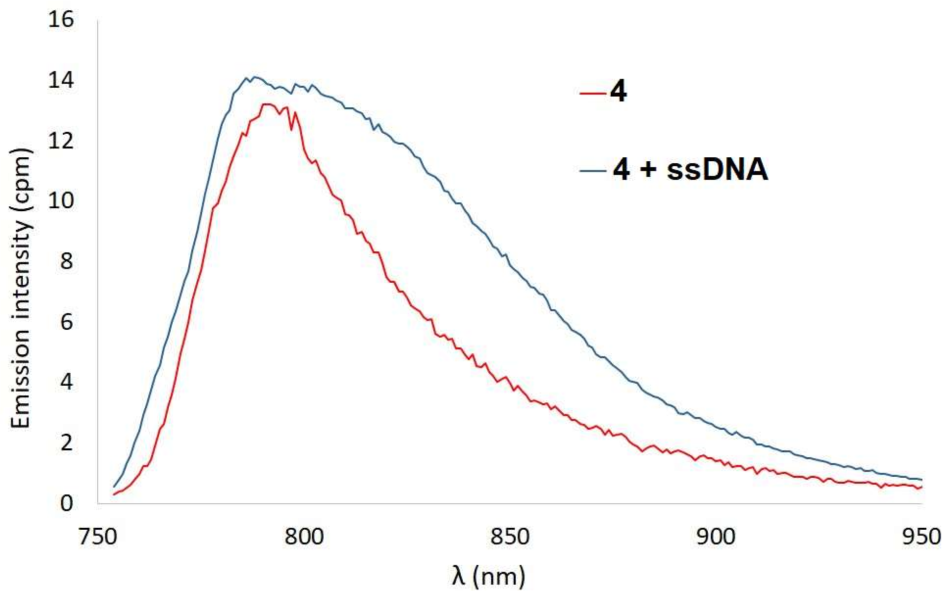

Figure S15. Emission profile of **4** in 9:1 Tris buffer (10 mM Tris-HCl, pH 7.5):DMSO in the absence and the presence of ssDNA (8 μM). λ_{ex} = 445 nm, [**4**] = 4 μM.

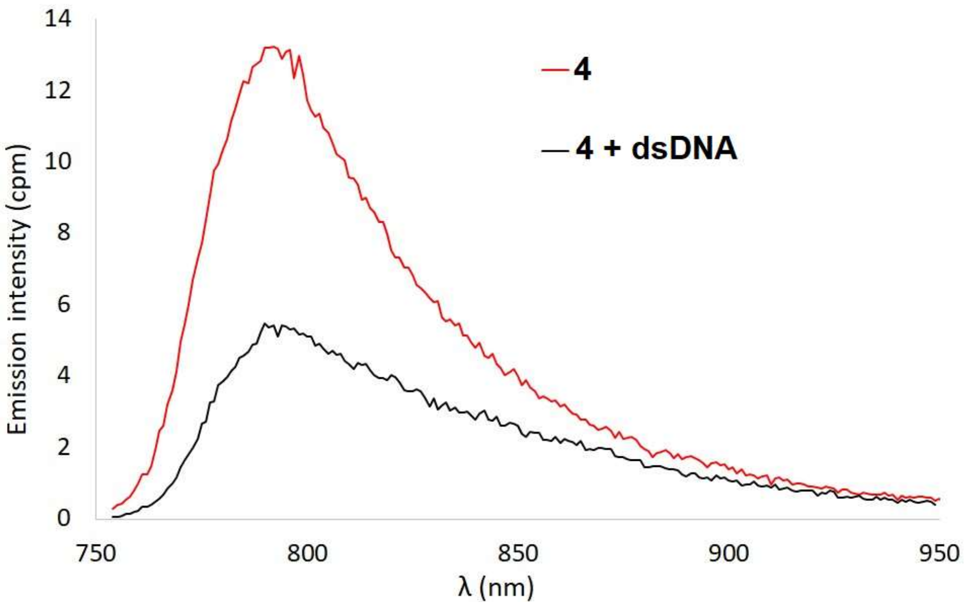

Figure S16. Emission profile of **4** in 9:1 Tris buffer (10 mM Tris-HCl, pH 7.5):DMSO in the absence and the presence of dsDNA (8 μM). λ_{ex} = 445 nm, [**4**] = 4 μM.

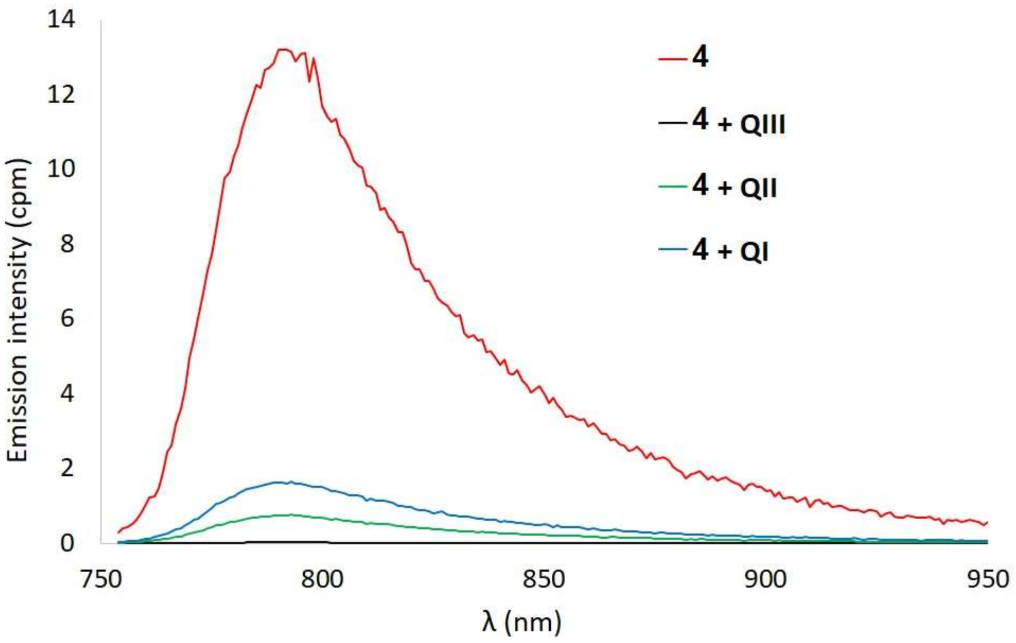

Figure S17. Emission profile of **4** in 9:1 Tris buffer (10 mM Tris-HCl, pH 7.5):DMSO in the absence and the presence of **QI**, **QII** and **QIII** (8 μM). λ_{ex} = 445 nm, [**4**] = 4 μM.

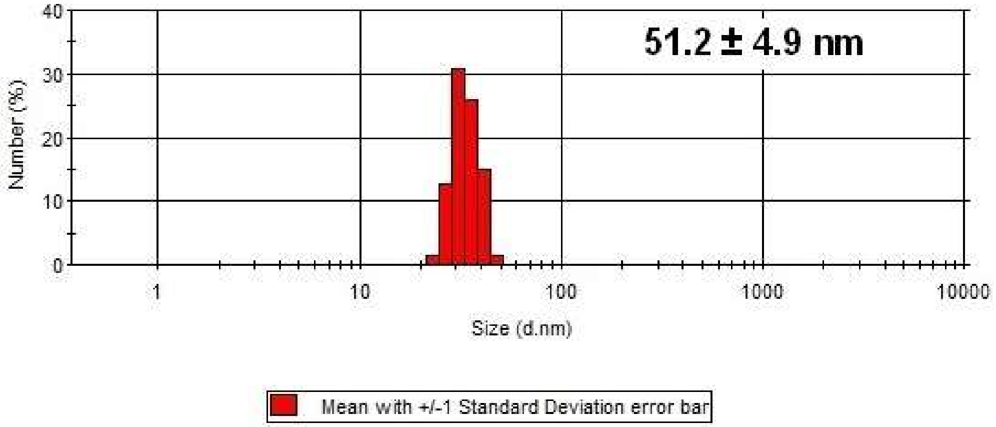

Figure S18. DLS particle size analysis of **4** (4 μM) in the presence of ssDNA (8 μM) in 9:1 Tris buffer (10 mM Tris-HCl, pH 7.5):DMSO.

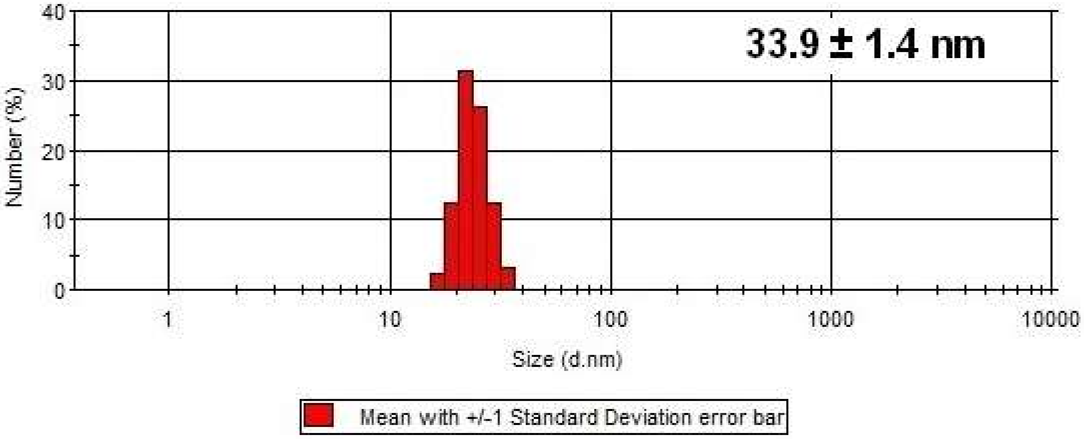

Figure S19. DLS particle size analysis of **4** (4 μM) in the presence of dsDNA (8 μM) in 9:1 Tris buffer (10 mM Tris-HCl, pH 7.5):DMSO.

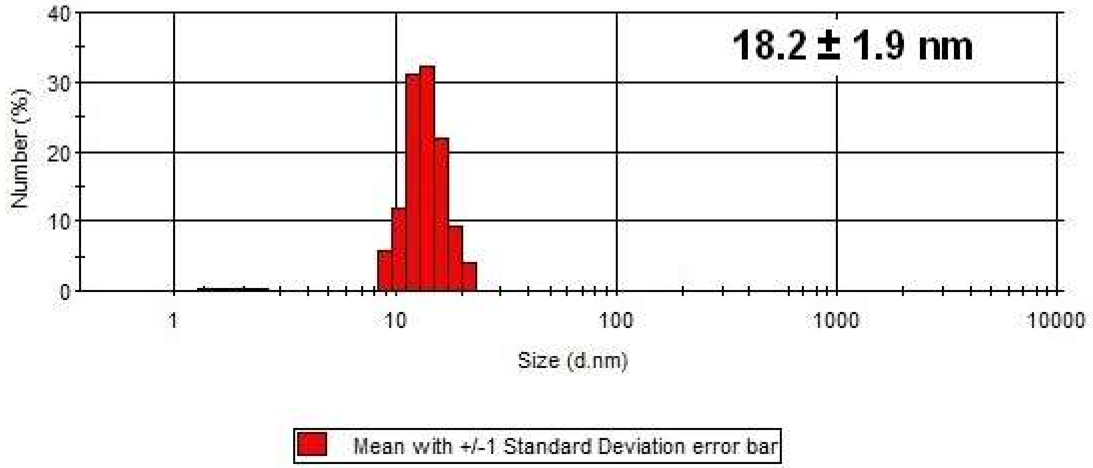

Figure S20. DLS particle size analysis of **4** (4 μM) in the presence of **QI** (8 μM) in 9:1 Tris buffer (10 mM Tris-HCl, pH 7.5):DMSO.

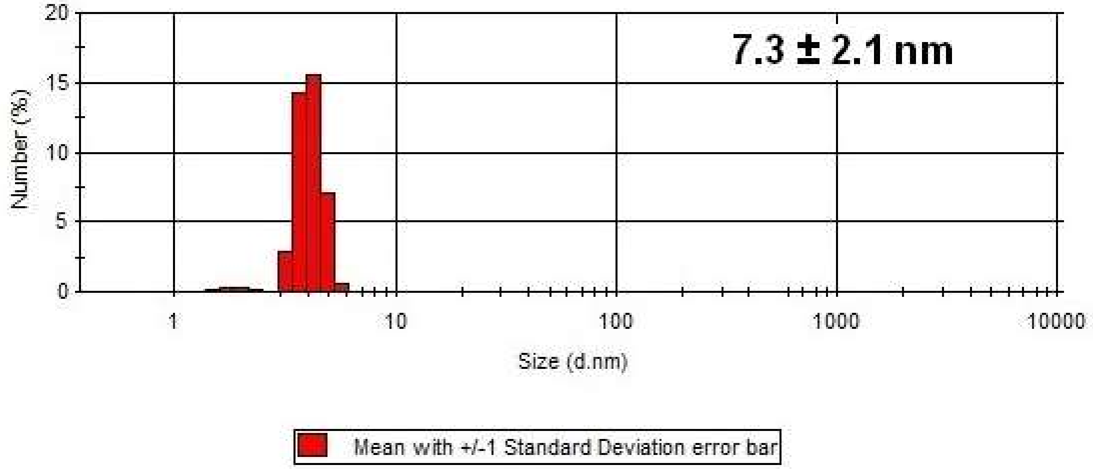

Figure S21. DLS particle size analysis of **4** (4 μM) in the presence of **QII** (8 μM) in 9:1 Tris buffer (10 mM Tris-HCl, pH 7.5):DMSO.

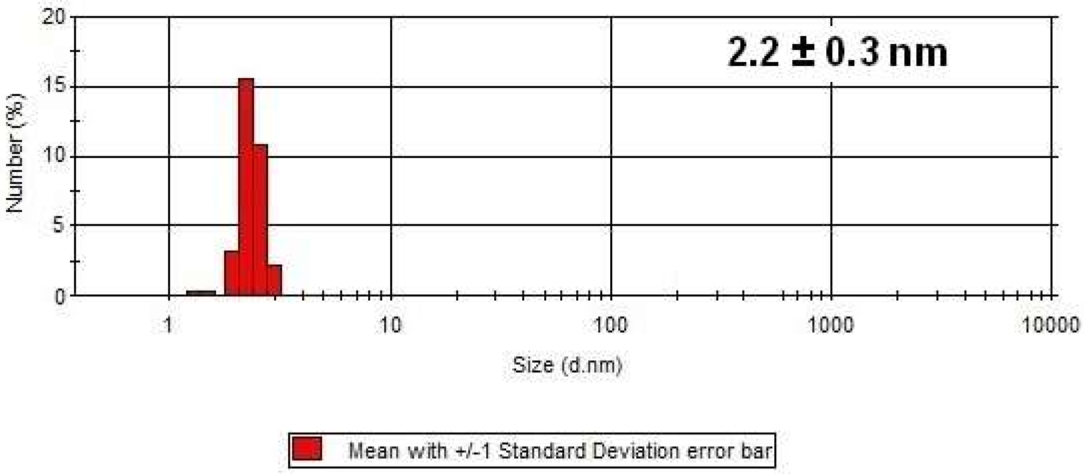

Figure S22. DLS particle size analysis of **4** (4 μM) in the presence of **QIII** (8 μM) in 9:1 Tris buffer (10 mM Tris-HCl, pH 7.5):DMSO.

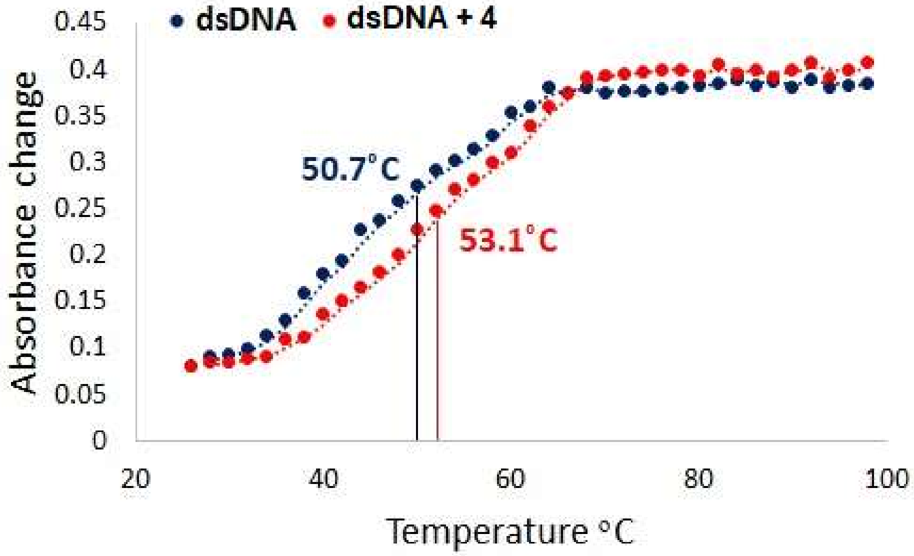

Figure S23. Changes in UV-vis absorbance at 260 nm of dsDNA (8 μM) with and without **4** (4 μM) in 9:1 Tris buffer (10 mM Tris-HCl, pH 7.5):DMSO upon increasing temperature.

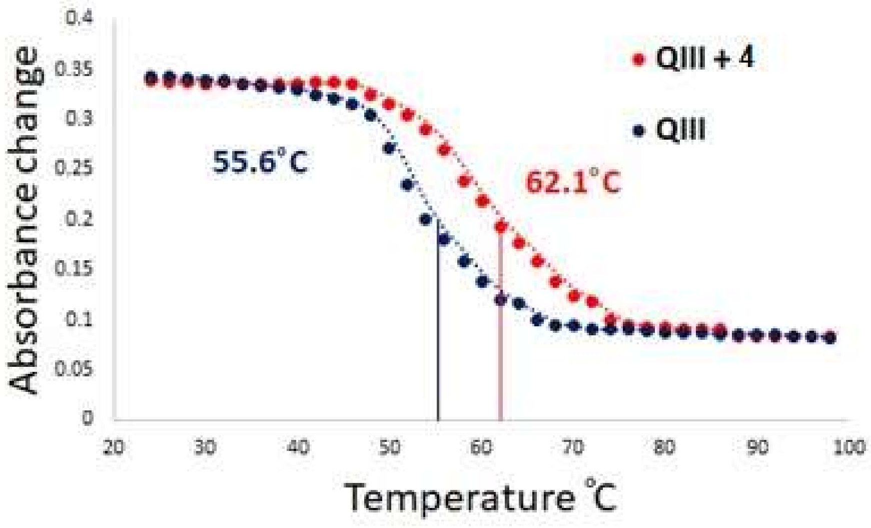

Figure S24. Changes in UV-vis absorbance at 295 nm of **QIII** (8 μM) with and without **4** (4 μM) in 9:1 Tris buffer (10 mM Tris-HCl, pH 7.5):DMSO upon increasing temperature.

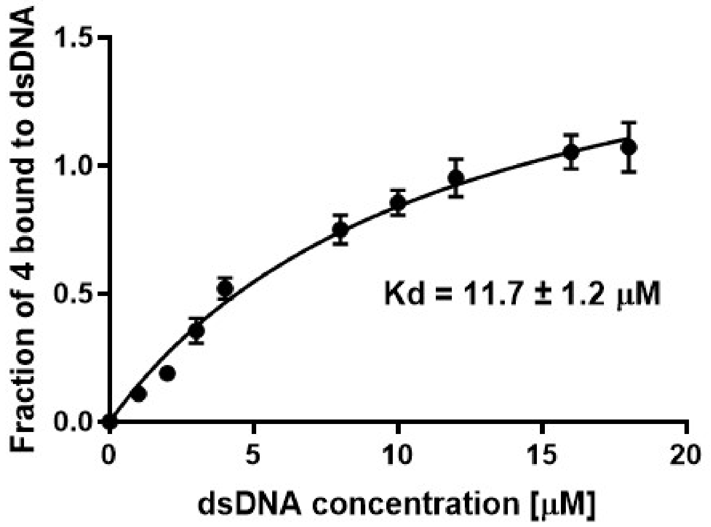

Figure S25. Saturation binding isotherm generated by GraphPad Prism using various concentrations of dsDNA (0-18 μM) towards **4** (4 μM).

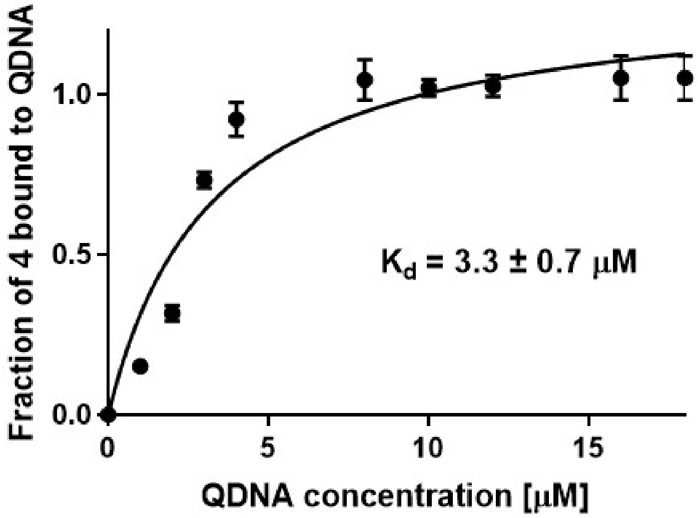

Figure S26. Saturation binding isotherm generated by GraphPad Prism using various concentrations of **QIII** (0-18 μM) towards **4** (4 μM).

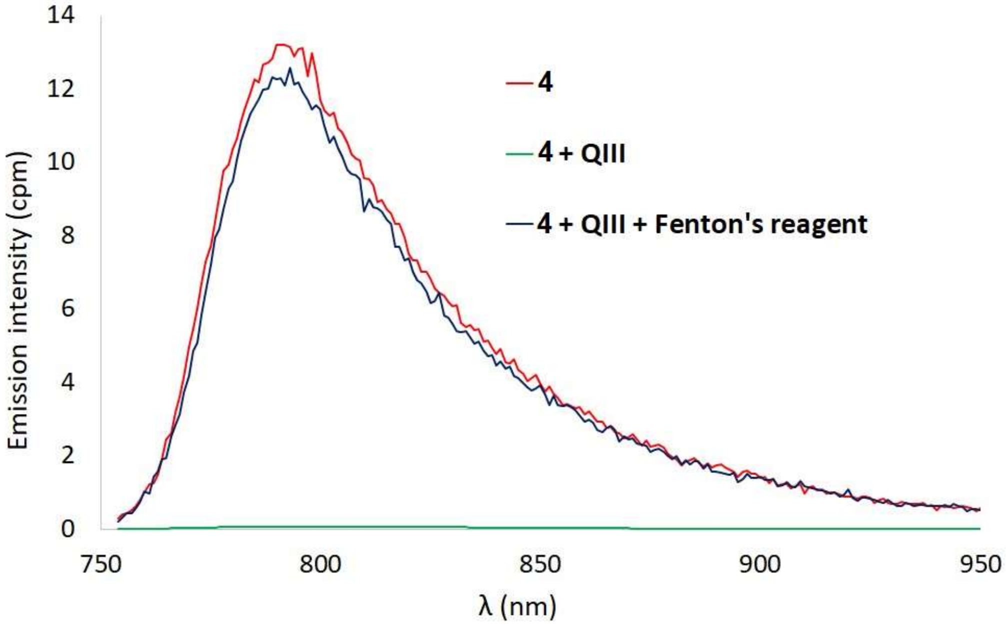

Figure S27. Emission profile of **4** and **4/QIII** in 9:1 Tris buffer (10 mM Tris-HCl, pH 7.5):DMSO in the absence and the presence of Fenton's reagent (1.4 mM $FeSO_4$ + 36 mM H_2O_2). λ_{ex} = 445 nm, [**4**] = 4 μM, **QIII** (8 μM).

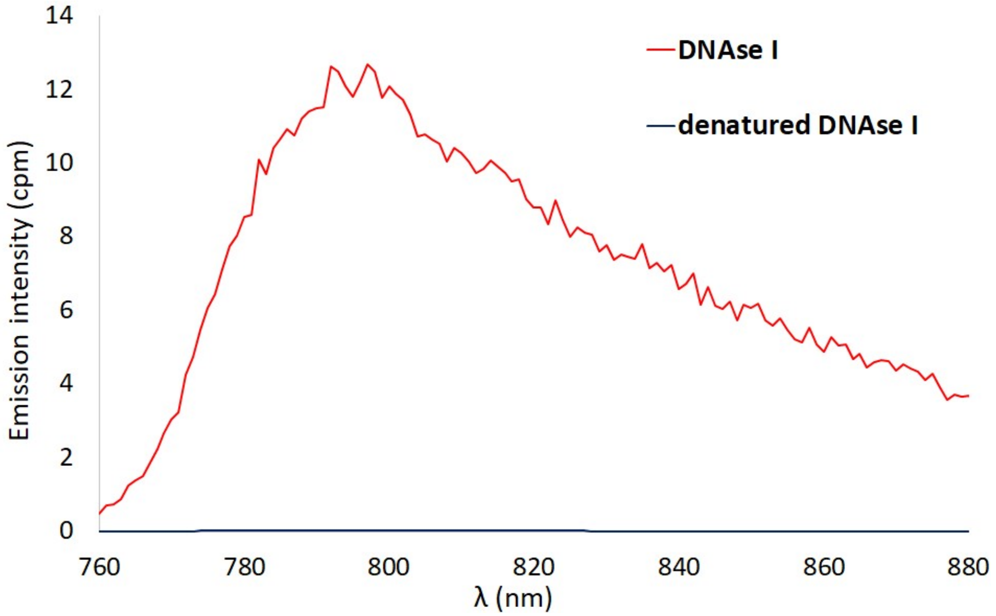

Figure S28. Emission profile of **4/QIII** ensemble in 9:1 Tris buffer (10 mM Tris-HCl, pH 7.5):DMSO in the presence of DNAse I (4 U/ml) and heat denatured DNAse I (4 U/ml) by heating at 65 °C for 10 min in the presence of 0.3 mM EDTA. λ_{ex} = 445 nm, [**4**] = 4 μM, **QIII** (8 μM). All measurements were done after incubation at room temperature for 10 min.

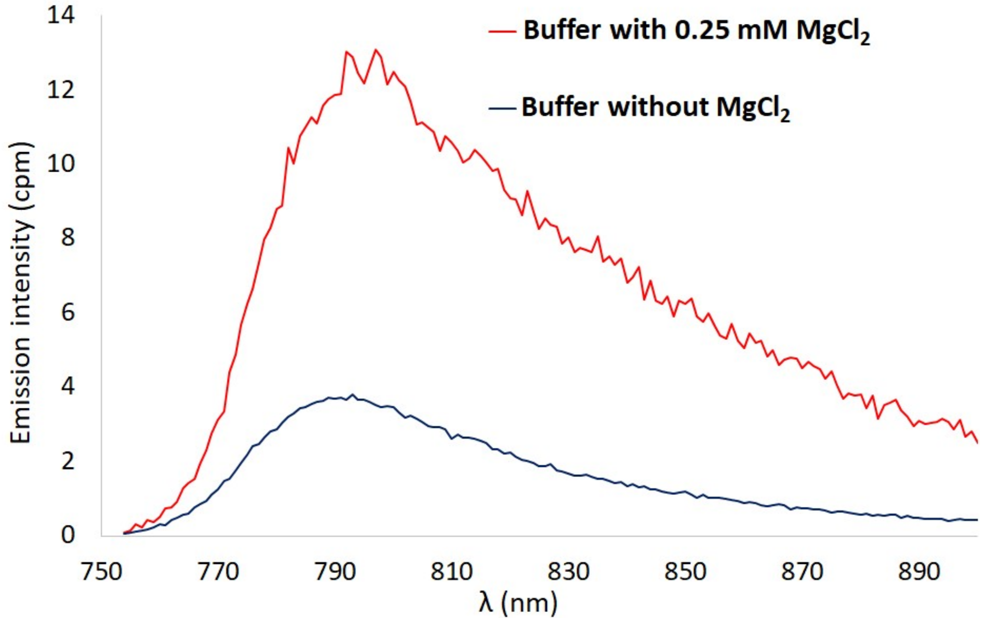

Figure S29. Emission profile of **4/QIII** ensemble in 9:1 Tris buffer (10 mM Tris-HCl, pH 7.5):DMSO in the presence of DNAse I (4 U/ml) using a reaction buffer with and without $MgCl_2$. λ_{ex} = 445 nm, [**4**] = 4 μM, **QIII** (8 μM). All measurements were done after incubation at room temperature for 10 min.

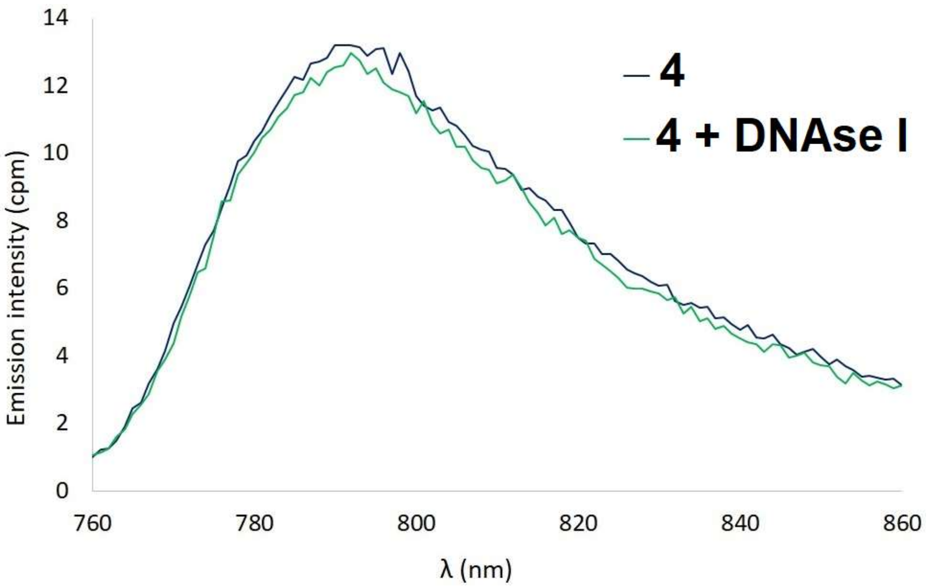

Figure S30. Emission profile of **4** in 9:1 Tris buffer (10 mM Tris-HCl, pH 7.5):DMSO in the presence and absence of DNAse I (4 U/ml). λ_{ex} = 445 nm, [**4**] = 4 μM. All measurements were done after incubation at room temperature for 10 min.

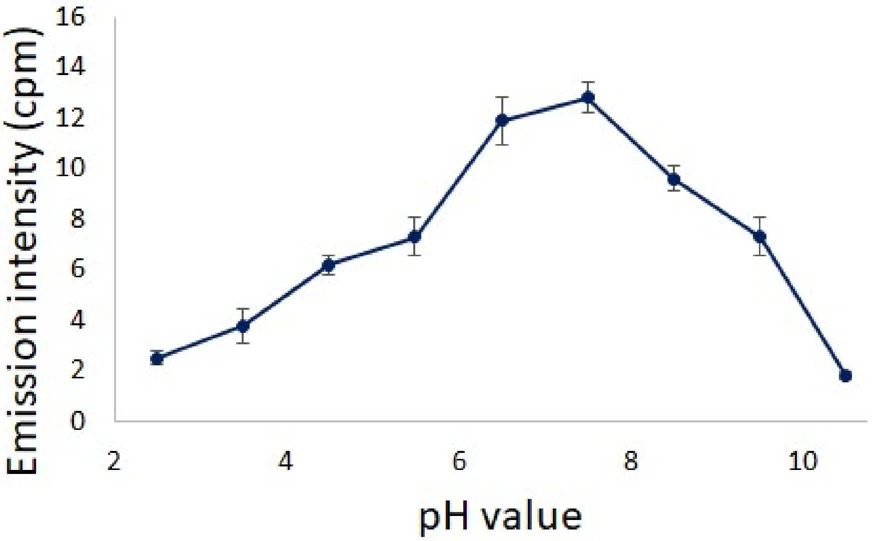

Figure S31. NIR Emission intensity of **4/QIII** ensemble in 9:1 Tris buffer (10 mM Tris-HCl):DMSO in the presence of DNAse I (4 U/ml) at different pH values. λ_{ex} = 445 nm, [**4**] = 4 μM, **QIII** (8 μM). Error bars represent standard deviation (n = 3).

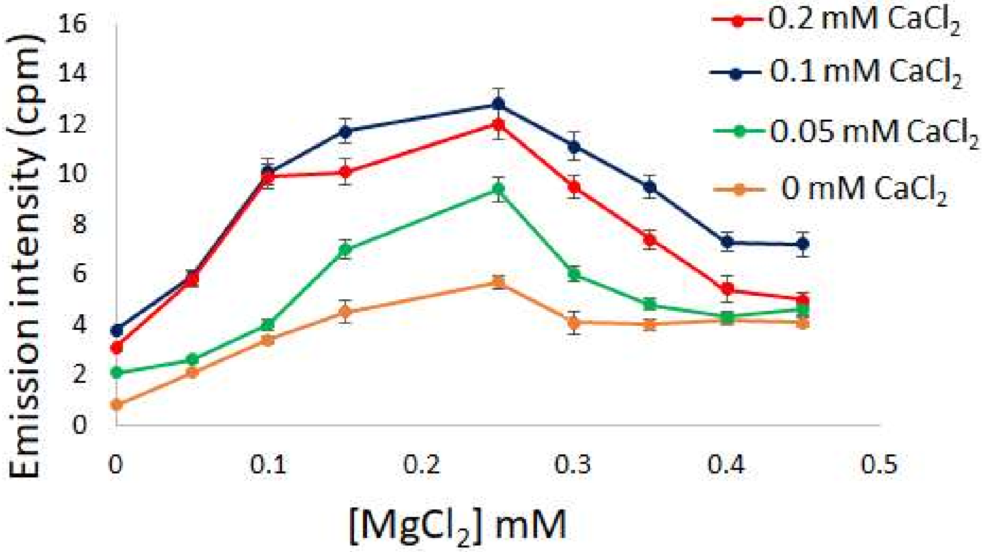

Figure S32. NIR Emission intensity of **4**/**QIII** ensemble in 9:1 Tris buffer (10 mM Tris-HCl, pH 7.5):DMSO in the presence of DNAse I (4 U/ml) at different combinations of $MgCl_2$ and $CaCl_2$. λ_{ex} = 445 nm, [**4**] = 4 μM, **QIII** (8 μM). Error bars represent standard deviation (n = 3).

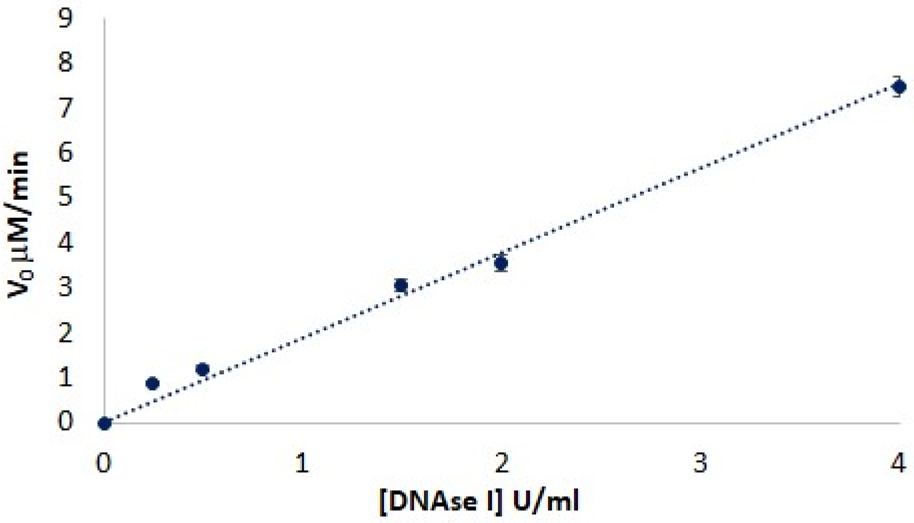

Figure S33. Initial cleavage velocity (V_0) of **4**/**QIII** ensemble as a function of DNAse I concentration.

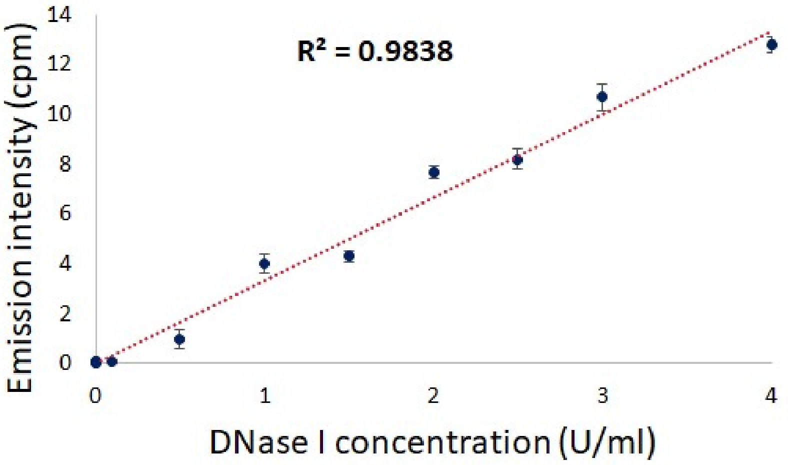

Figure S34. The linear correlation of NIR emission intensity of **4**/**QIII** ensemble and DNAse I concentrations in human serum samples. λ_{ex} = 445 nm, [**4**] = 4 μM, **QIII** (8 μM). Error bars represent standard deviation (n = 3).

Table S1. Comparison of the developed DNAse I assay to other fluorescence-based DNAse I assays.

Method	Limit of detection	Detection time (min)	Labeling	Signaling	Multiwell plate format
Graphene-based fluorescent assay[8]	1.75 unit/ml	8	Labelling is required	Turn-on	No
Graphene-based fluorescent assay[9]	1 unit/ml	10	Labelling is required	Turn-on	No
Self-quenched fluorescent reporter DNA[10]	Not determined	25	Labelling is required	Turn-on	Yes
Nano-graphene-based fluorescent assay[11]	0.005 unit/ml	40	Labelling is required	Turn-on	No
Malachite green dye/G-quadruplex[12]	1 unit/ml	180	Label-free	Turn-off	No
DNA-templated gold/silver nanoclusters[13]	1.5 unit/ml	30	Label-free	Turn-off	No
DNA-templated silver nanocluster/graphene oxide nanocomposite[14]	0.1 unit/ml	150	Label-free	Turn-on	Yes
dsDNA coupled with PicoGreen[15]	5 pg	60	Label-free	Turn-on	Yes
FRET dsDNA probe[16]	40 unit/L	20	Labelling is required	Turn-on	No
DNAseAlert™ QC system[17]	0.005 unit	30-60	Labelling is required	Turn-on	Yes
This work	0.002 unit/ml	10	Label-free	Turn-on	Yes

Table S2. Determination of DNAse I activity in diluted human serum.

Added DNAse I (U/ml)	Measured DNAse I[a] (U/ml)	Coefficient of variation[c] (%)	% Recovery[d]
**0.15**	0.16 (0.003)[b]	1.87	106
**0.5**	0.48 (0.01)	2.08	96
**1**	1.09 (0.04)	3.66	109
**4**	3.88 (0.07)	1.8	97

[a]Mean value of three measurements. All readings were subtracted from a blank human serum sample which accounts for endogenous DNAse I activity.

[b]Standard deviation (SD) for measured DNAse I (n=3).

[c]Coefficient of variation = SD/mean ×100.

[d]% Recovery = Measured DNAse I/Added DNase I ×100.

Table S3. Determination of IC_{50} values of known DNAse I inhibitors.

DNAse I inhibitor	IC_{50} (µM)	Reported IC_{50} (µM)[10]
**EDTA**	202 ± 3.2	190
**JR-132 (1,4-phenylene-bis-aminoguanidine hydrochloride)[18]**	2.29 ± 0.2	2.73
**$ZnCl_2$**	20.7 ± 0.4	18

References:

1. Ang, D. L.; Harper, B. W. J.; Cubo, L.; Mendoza, O.; Vilar, R.; Aldrich-Wright, J. *Chem. Eur. J.* **2016**, *22*, 2317-2325.
2. Gabr M. T.; Pigge, F. C. *Inorg. Chem.* **2018**, *57*, 12641-12649.
3. Gros, P.; Fort, Y. *J. Org. Chem.* **2003**, 63, 2028-2029.
4. Barnes, J. C.; Juríček, M.; Strutt, N. L.; Frasconi, M.; Sampath, S.; Giesener, M. A.; McGrier, P. L.; Bruns, C. J.; Stern, C. L.; Sarjeant, A. A.; Stoddart, J. F. *J. Am. Chem. Soc.* **2013**, *135*, 183-192.
5. Petraccone, L.; Fotticchia, I.; Cummaro, A.; Pagano, B.; Ginnari-Satriani, L.; Haider, S.; Randazzo, A.; Novellino, E.; Neidle S.; Giancola, C. *Biochimie* **2011**, *93*, 1318-1327.
6. Hein, P.; Michel, M. C.; Leineweber, K.; Wieland, T.; Wettschureck N.; Offermanns, S. Receptor and Binding Studies in Practical Methods in Cardiovascular Research (Eds.: S. Dhein, F. W. Mohr, M. Delmar), Springer, Berlin, Heidelberg, 2005, pp. 723–783.
7. Zhang, J.-H.; Chung, T. D. Y.; Oldenburg, K. R. *J. Biomol. Screen.* **1999**, *4*, 67-73.
8. Zhou, Z.; Zhu, C.; Ren, J.; Dong, S. *Anal. Chim. Acta* **2012**, *740*, 88-92.
9. Xu, W.; Xie, Z.; Tong, C.; Peng, L.; Xiao, C.; Liu, X.; Zhu, Y.; Liu, B. *Anal. Bioanal. Chem.* **2016**, ***408***, 3801-3809.
10. Jang, D. S.; Penthala, N. R.; Apostolov, E. O.; Wang, X.; Fahmi, F.; Crooks P. A.; Basnakian, A. G. *J. Biomol. Screen.* **2015**, *20*, 202-211.
11. Zhao, C.; Chen, Y.; Fang, J.; Fan, J.; Tong, C.; Liu, X.; Liu B.; Wang, W. *RSC Adv.* 2017, **7**, 30911-30918.
12. Sun, S.-K.; Wang B.-B.; Yan, X.-P. *Analyst* **2013**, *138*, 2592-2597.
13. Dou, Y.; Yang, X. *Anal. Chim. Acta* **2013**, *784*, 53-58.
14. Lee, C. Y.; Park, K. S.; Jung Y. K.; Park, H. G. *Biosens. Bioelectron.* **2017**, *93*, 293-297.
15. Choi; S. J.; Szoka, F. C. *Anal. Biochem.* **2000**, *281*, 95-97.
16. Su, X.; Zhang, C.; Zhu, X.; Fang, S.; Weng, R.; Xiao, X.; Zhao, M. *Anal. Chem.* **2013**, *85*, 9939-9946.
17. DNAseAlert^{TM} QC system. https://www.thermofisher.com/order/catalog/product/AM1970 (Accessed on January 27, 2019).
18. Ring, J. R.; Zheng, F.; Haubner, A. J.; Littleton J. M.; Crooks, P. A. *Bioorg. Med. Chem.* **2013**, *21*, 1764-1774.

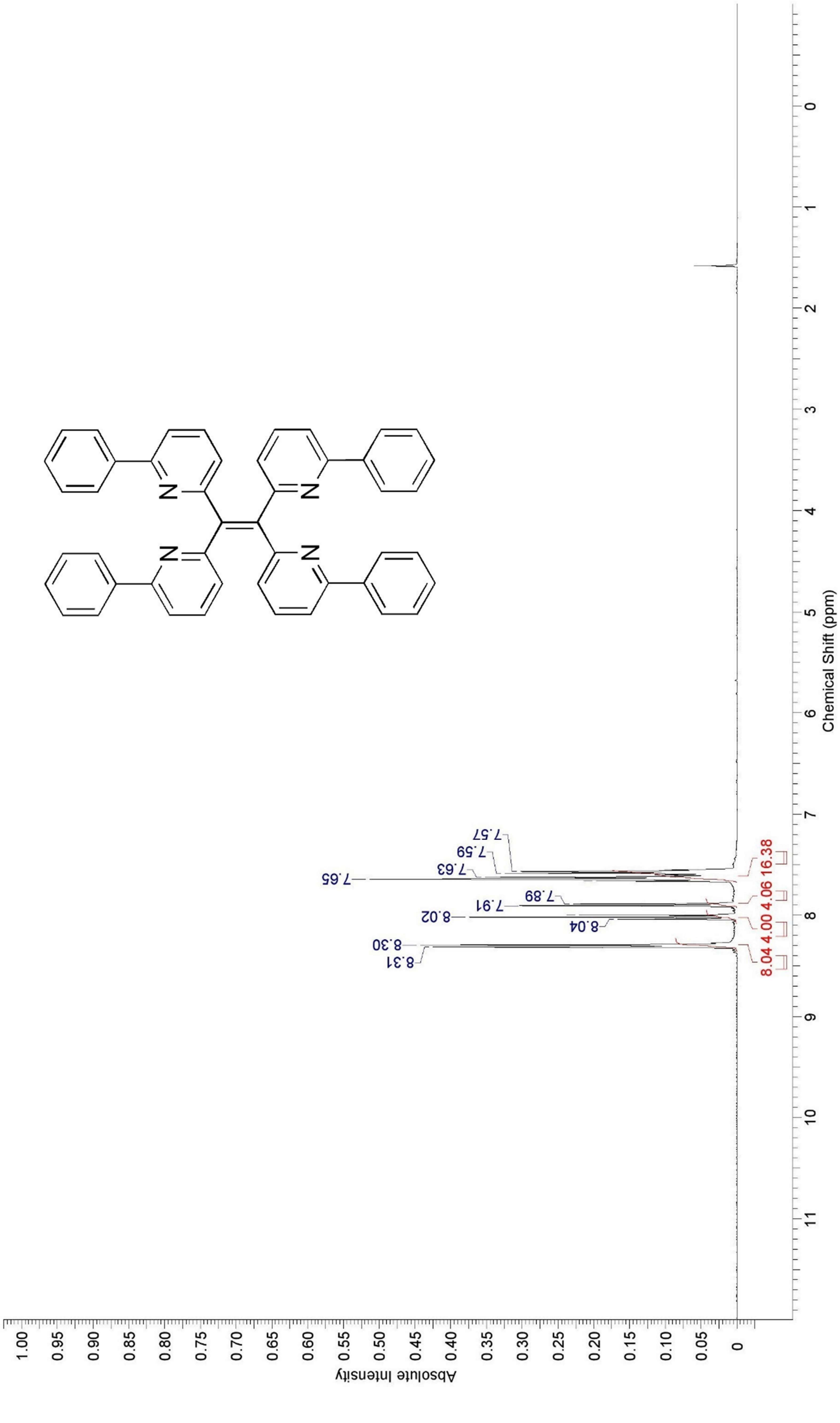

8.31
8.30
8.04
8.02
7.91
7.89
7.65
7.63
7.59
7.57
8.04 4.00 4.06 16.38
Chemical Shift (ppm)
Absolute Intensity

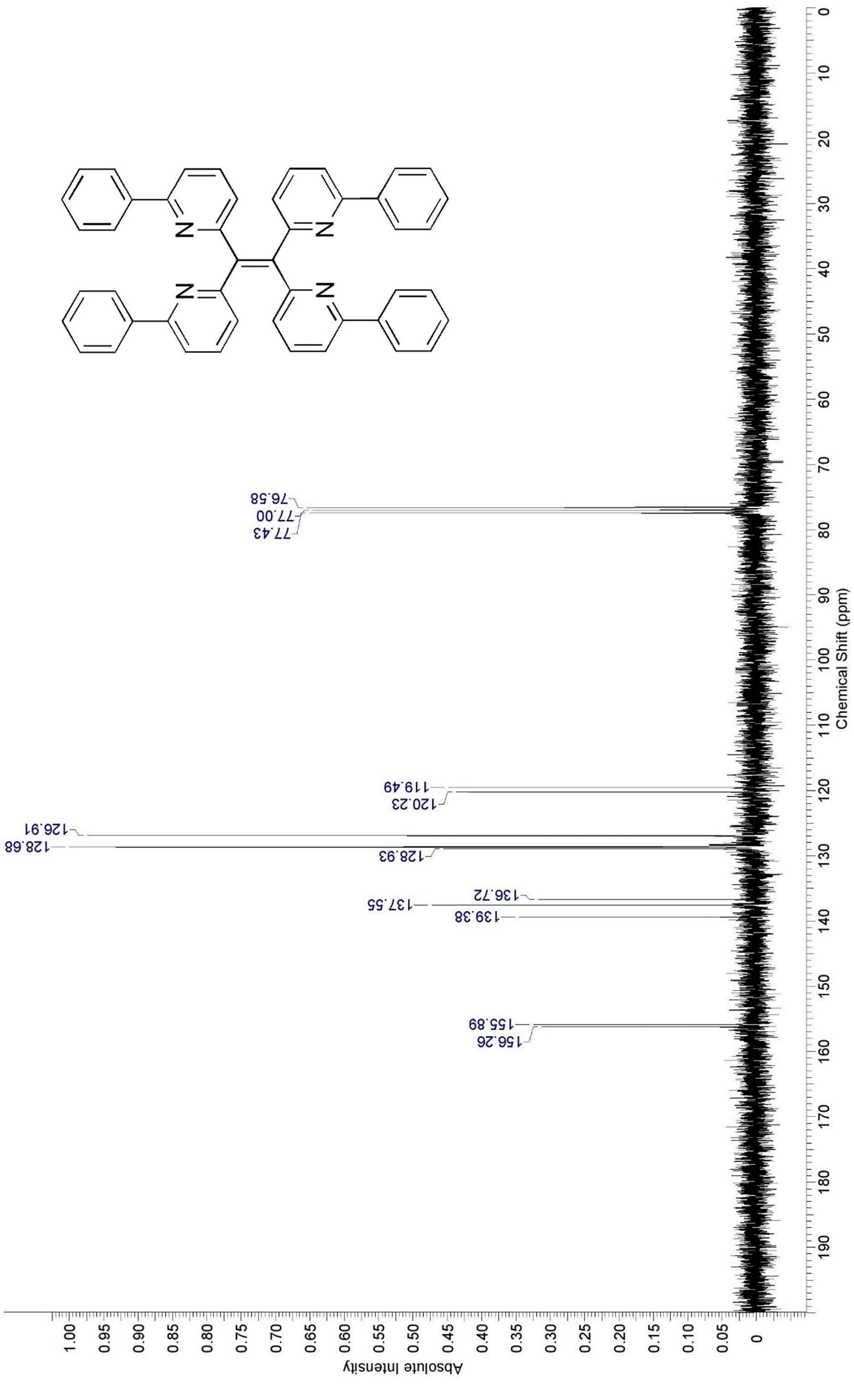
76.58
77.00
77.43
119.49
120.23
126.91
128.68
128.93
136.72
137.55
139.38
155.89
156.26
Chemical Shift (ppm)
Absolute Intensity

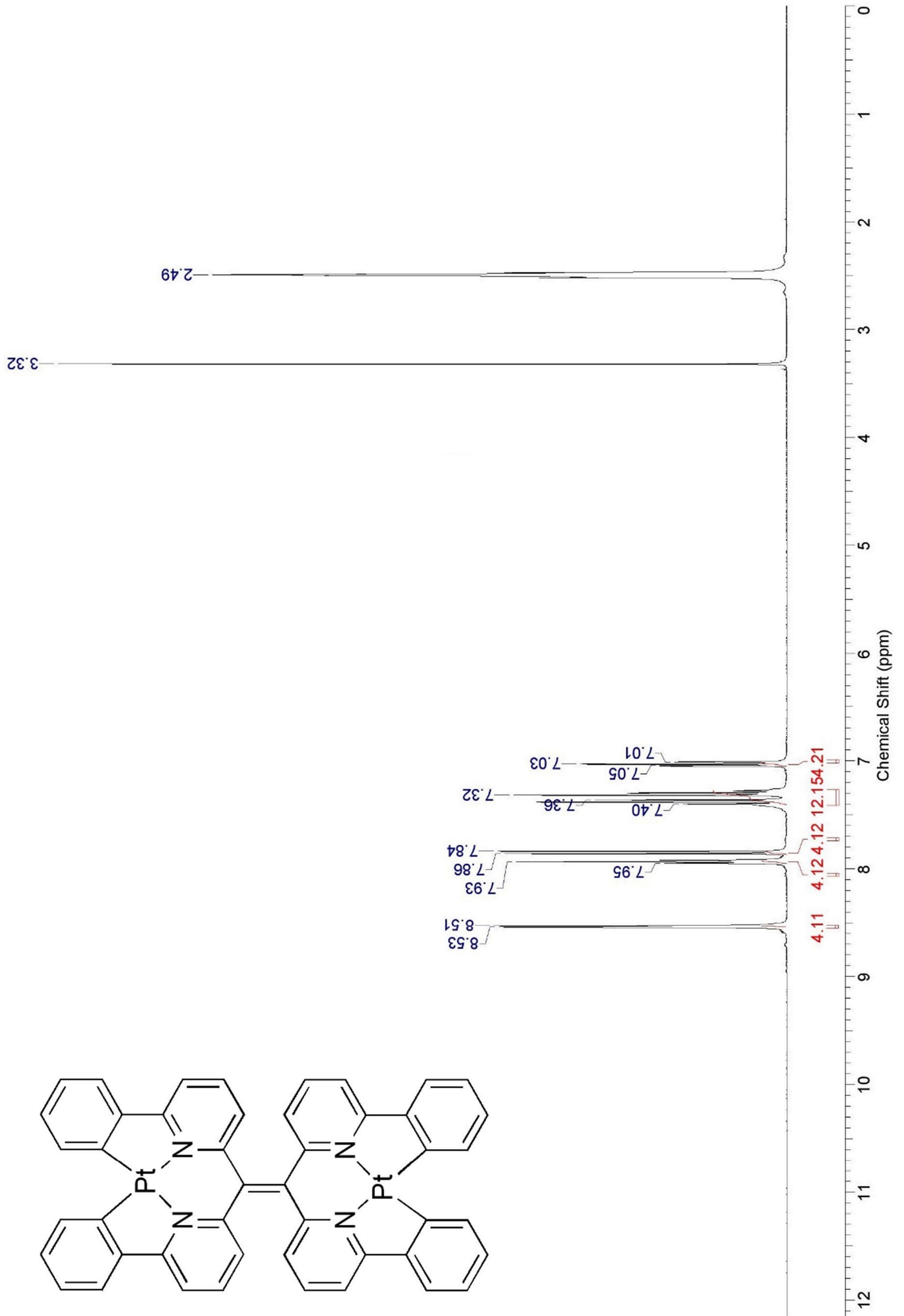

Chemical Shift (ppm)
Absolute Intensity
8.53
8.51
7.93
7.86
7.84
7.95
7.32
7.36
7.40
7.03
7.05
7.01
3.32
2.49
4.11
4.12
4.12
12.15
4.21
Pt
N

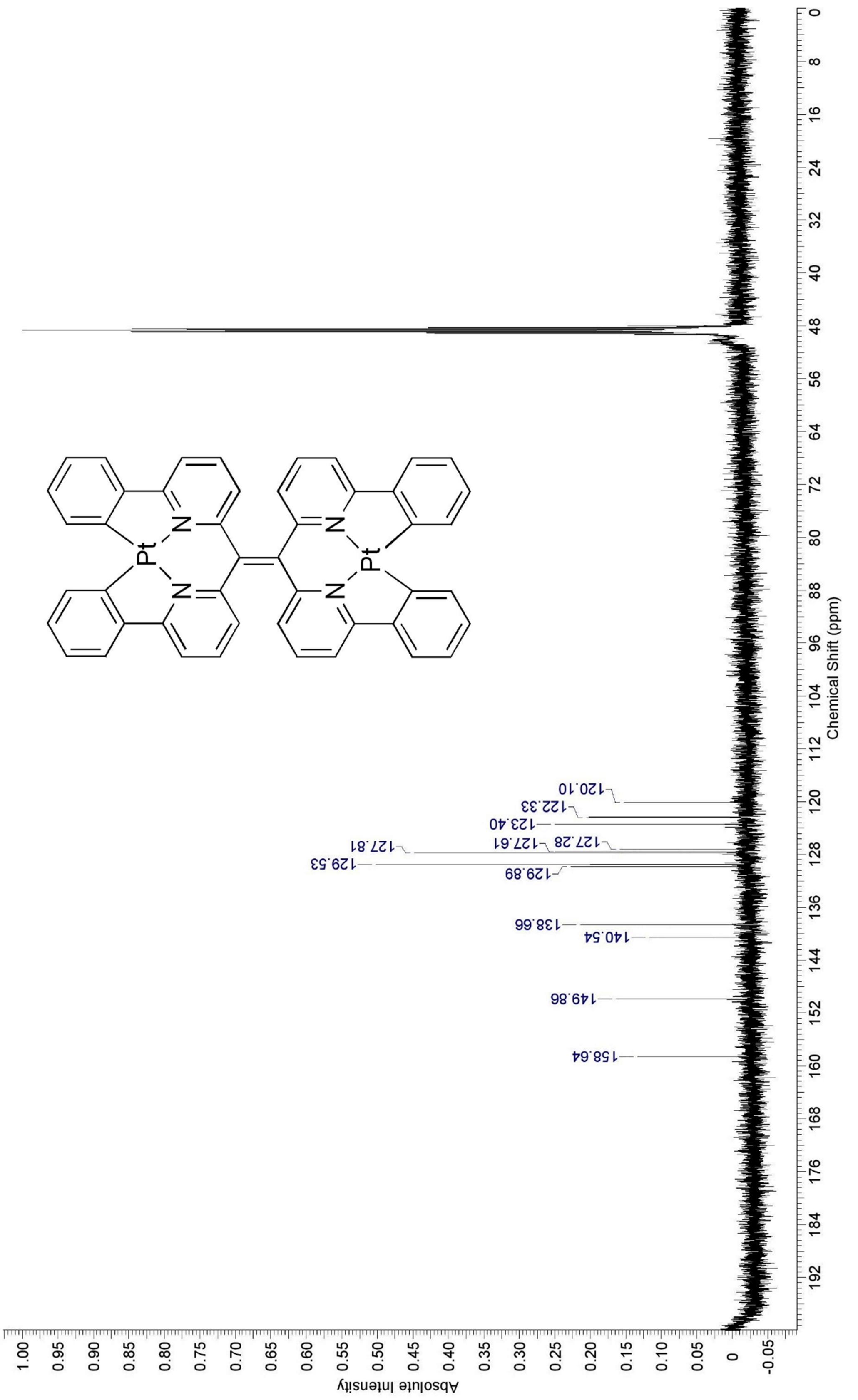

120.10
122.33
123.40
127.28
127.61
127.81
129.53
129.89
138.66
140.54
149.86
158.64
Chemical Shift (ppm)
Absolute Intensity
Pt
N

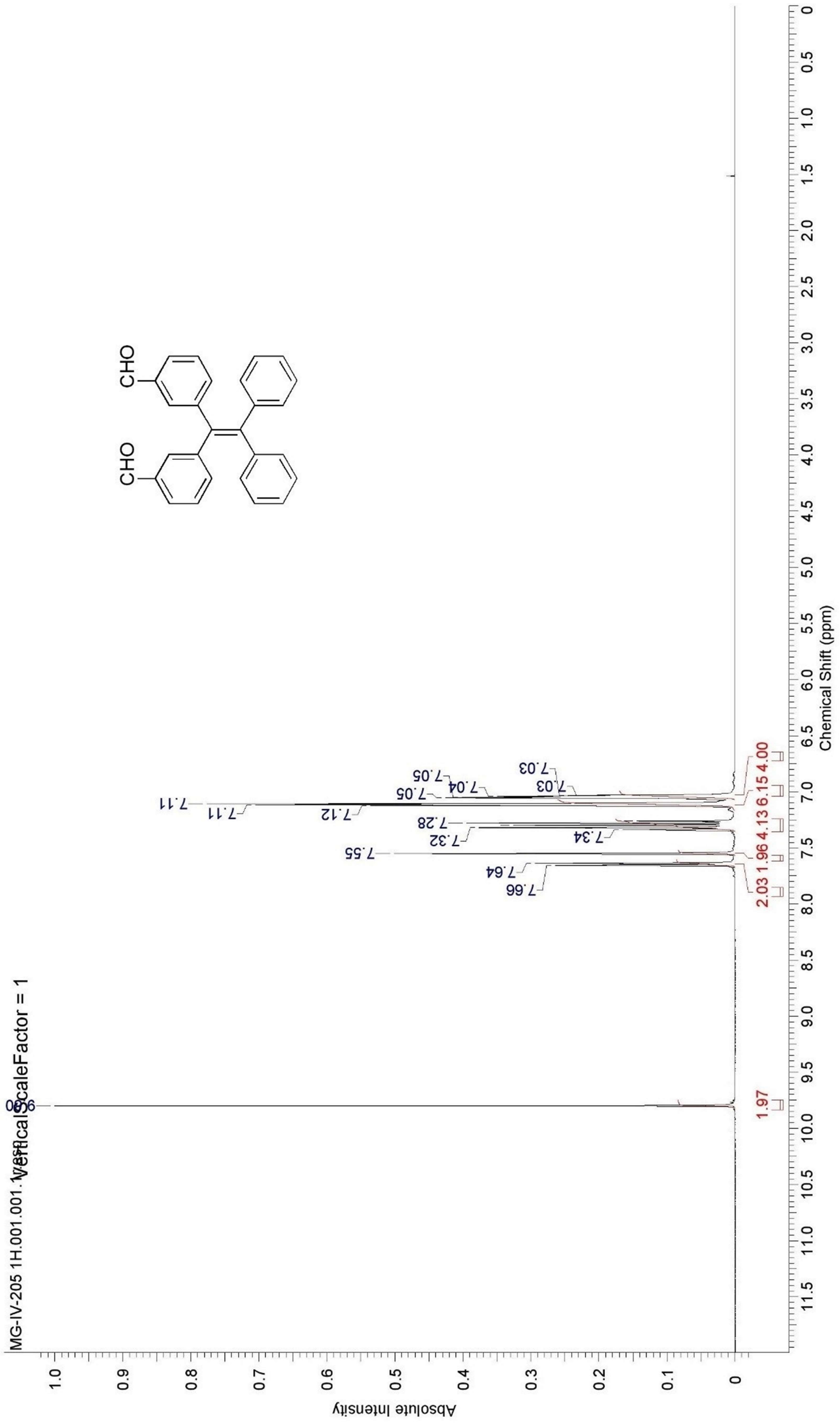

MG-IV-205 1H.001.001.1r.esp
VerticalScaleFactor = 1
CHO
CHO
9.86
7.66
7.64
7.55
7.34
7.32
7.28
7.12
7.11
7.11
7.05
7.05
7.04
7.03
7.03
1.97
2.03
1.96
4.13
6.15
4.00
Chemical Shift (ppm)
Absolute Intensity

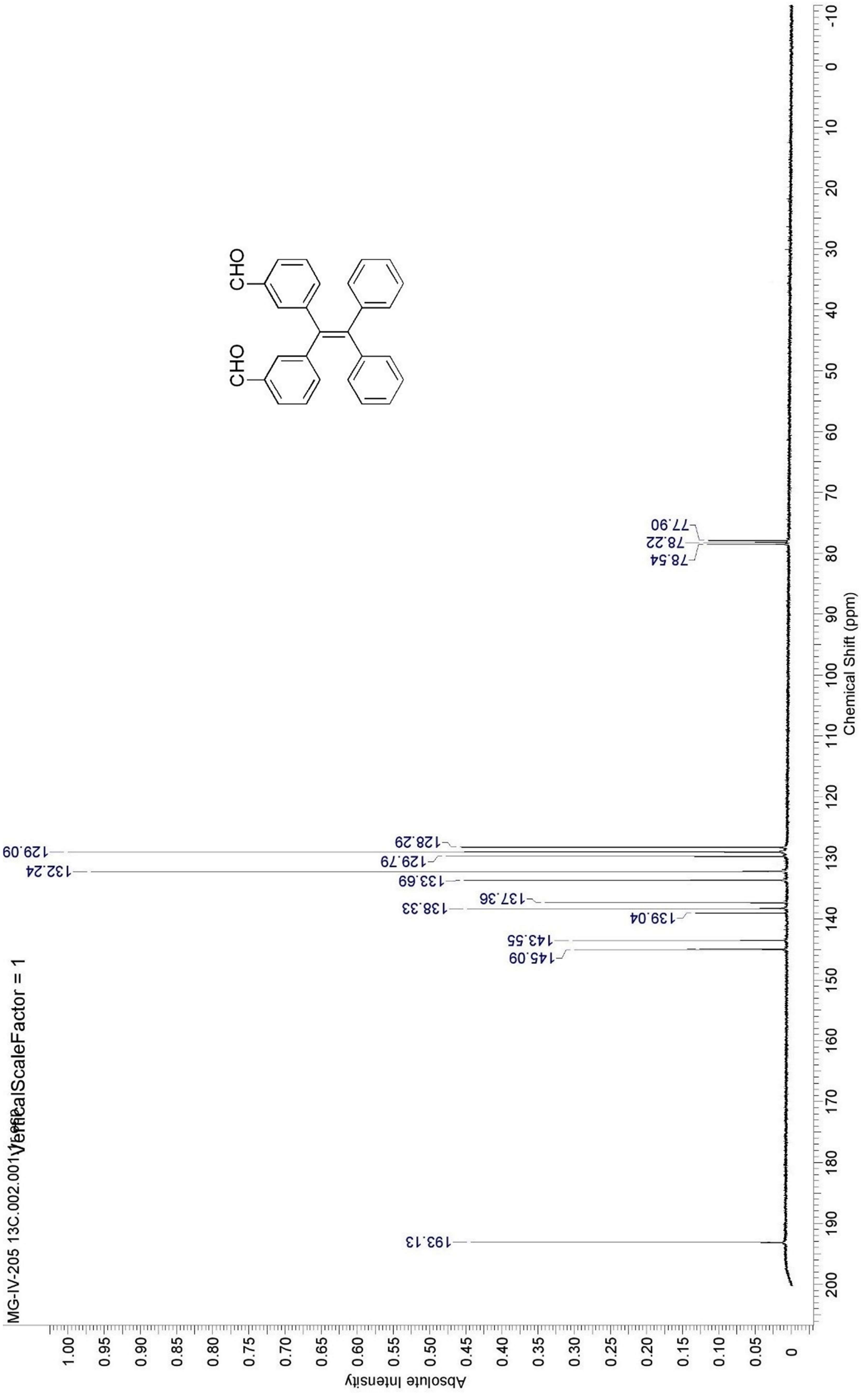
MG-IV-205 13C.002.001.1r.esp
VerticalScaleFactor = 1
CHO
CHO
193.13
145.09
143.55
139.04
138.33
137.36
133.69
132.24
129.79
129.09
128.29
78.54
78.22
77.90
Chemical Shift (ppm)
Absolute Intensity

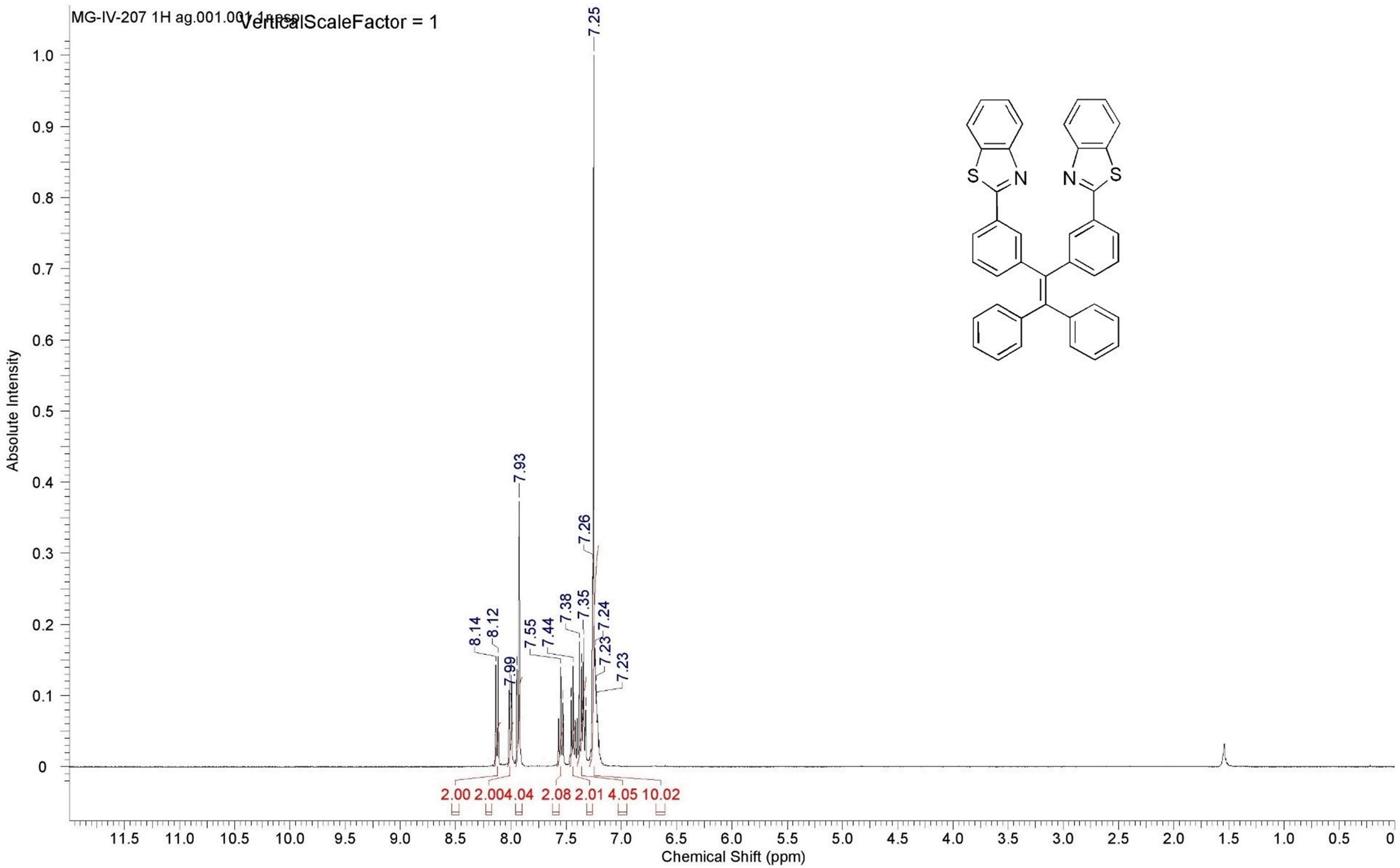
MG-IV-207 1H ag.001.001.1r.esp
VerticalScaleFactor = 1
7.25
7.93
7.26
8.14
8.12
7.99
7.55
7.44
7.38
7.35
7.24
7.23
7.23
2.00
2.00
4.04
2.08
2.01
4.05
10.02
Absolute Intensity
Chemical Shift (ppm)
S
N
N
S

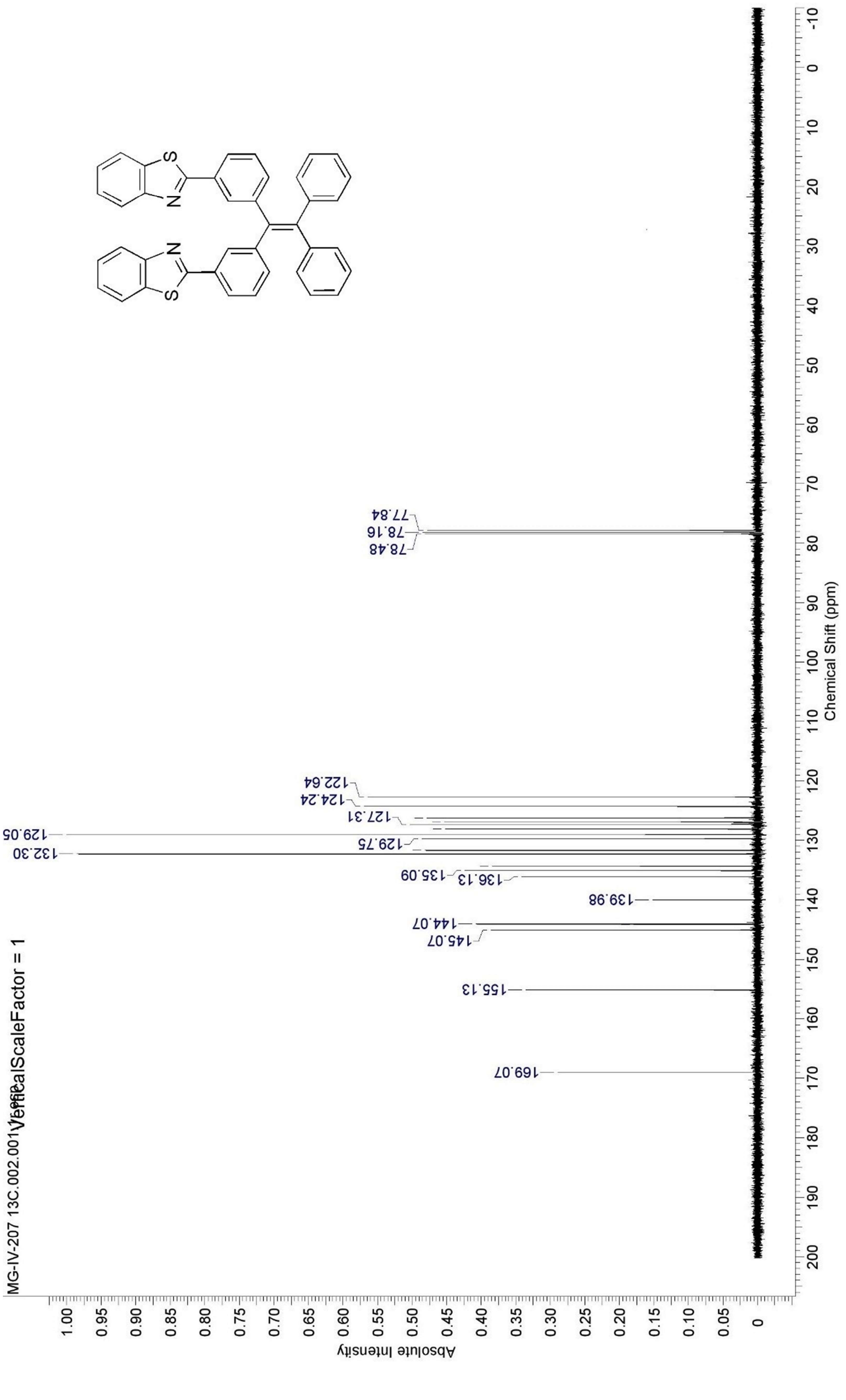

MG-IV-207 13C.002.001.1r.esp
VerticalScaleFactor = 1
169.07
155.13
145.07
144.07
139.98
136.13
135.09
132.30
129.75
129.05
127.31
124.24
122.64
78.48
78.16
77.84
Chemical Shift (ppm)
Absolute Intensity
S
N

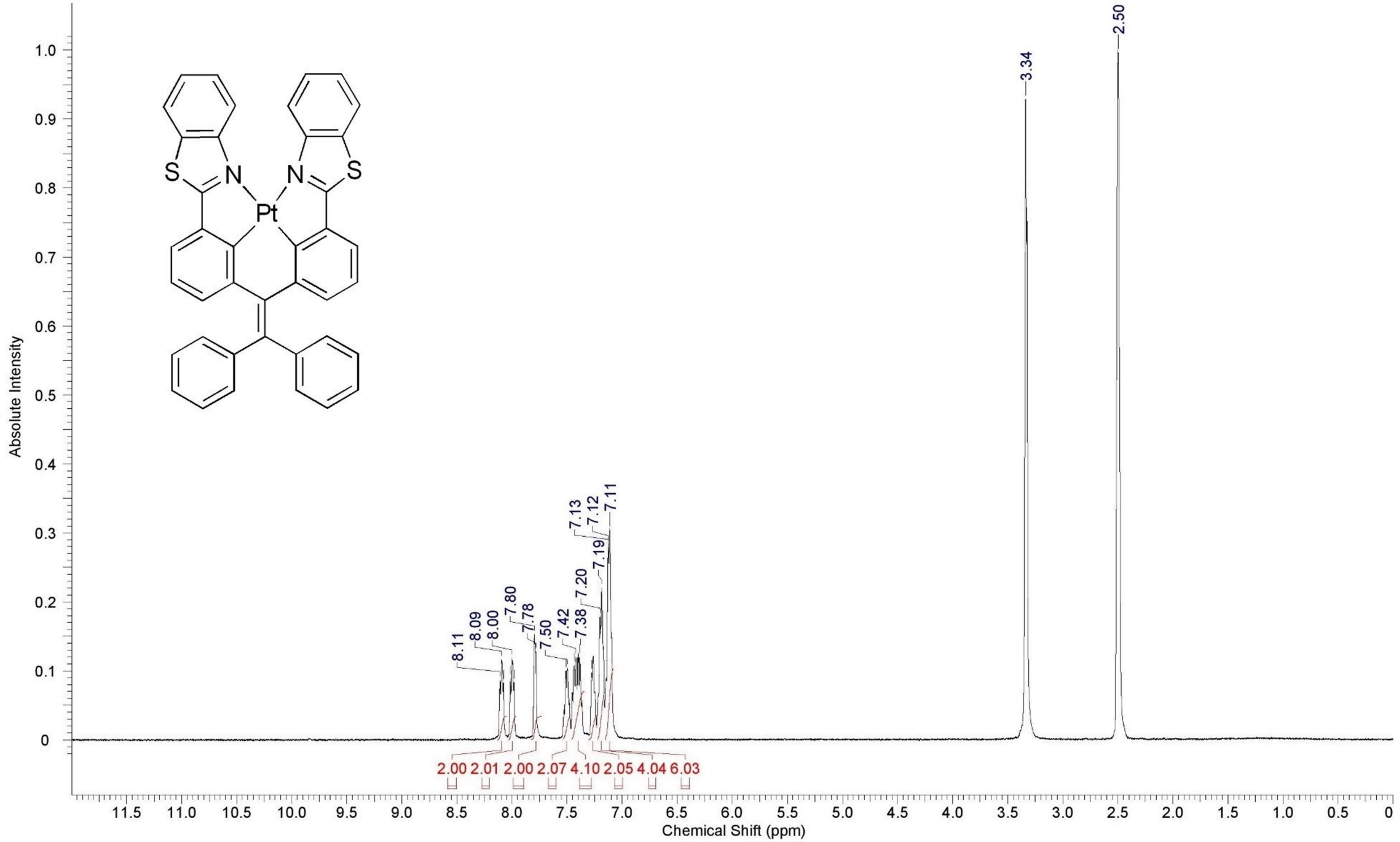
S
N
Pt
N
S
2.50
3.34
8.11
8.09
8.00
7.80
7.78
7.50
7.42
7.38
7.20
7.19
7.13
7.12
7.11
2.00 2.01 2.00 2.07 4.10 2.05 4.04 6.03
Absolute Intensity
Chemical Shift (ppm)

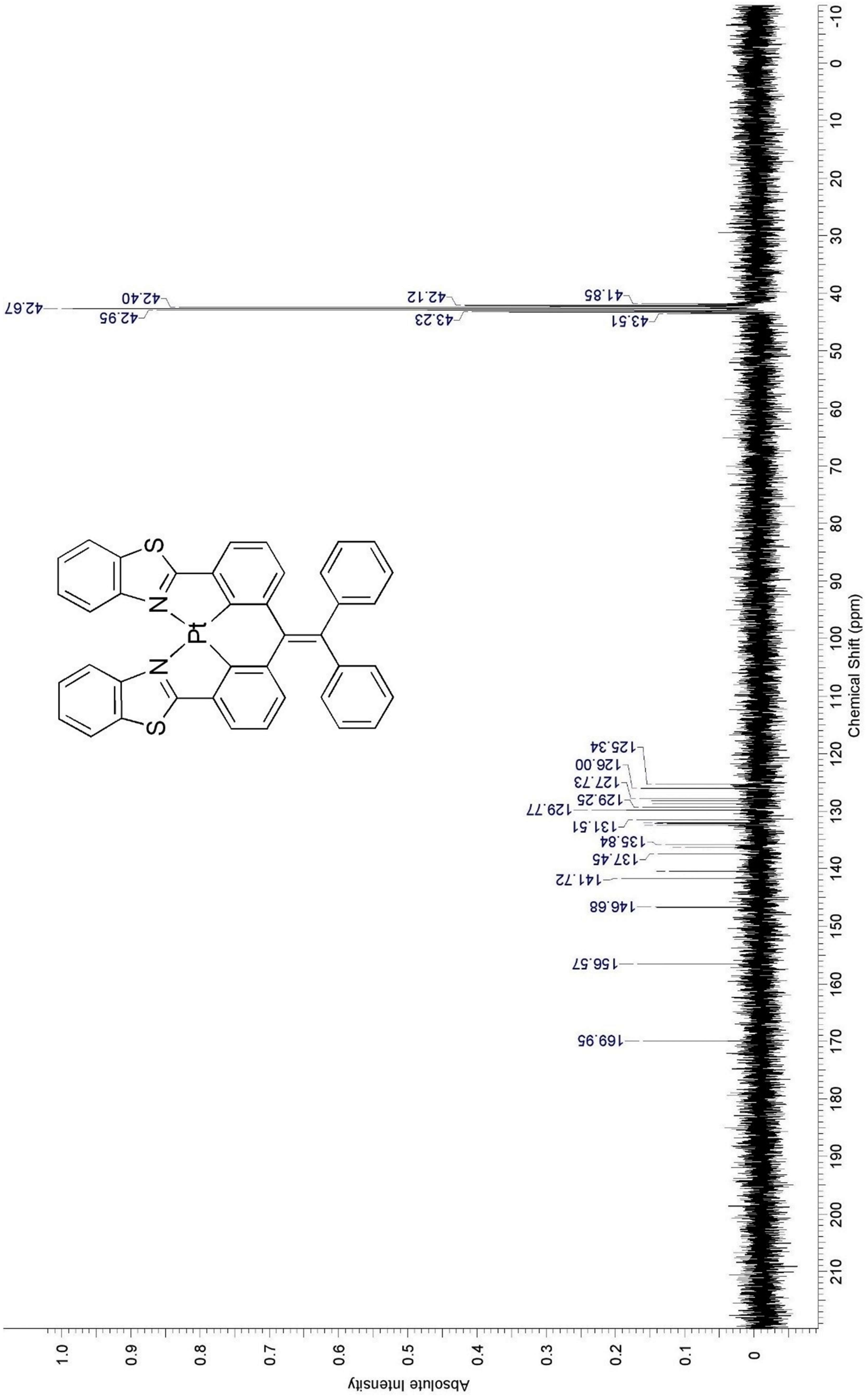

42.67
42.40
42.95
42.12
43.23
41.85
43.51
125.34
126.00
127.73
129.25
129.77
131.51
135.84
137.45
141.72
146.68
156.57
169.95
S
N
Pt
Chemical Shift (ppm)
Absolute Intensity

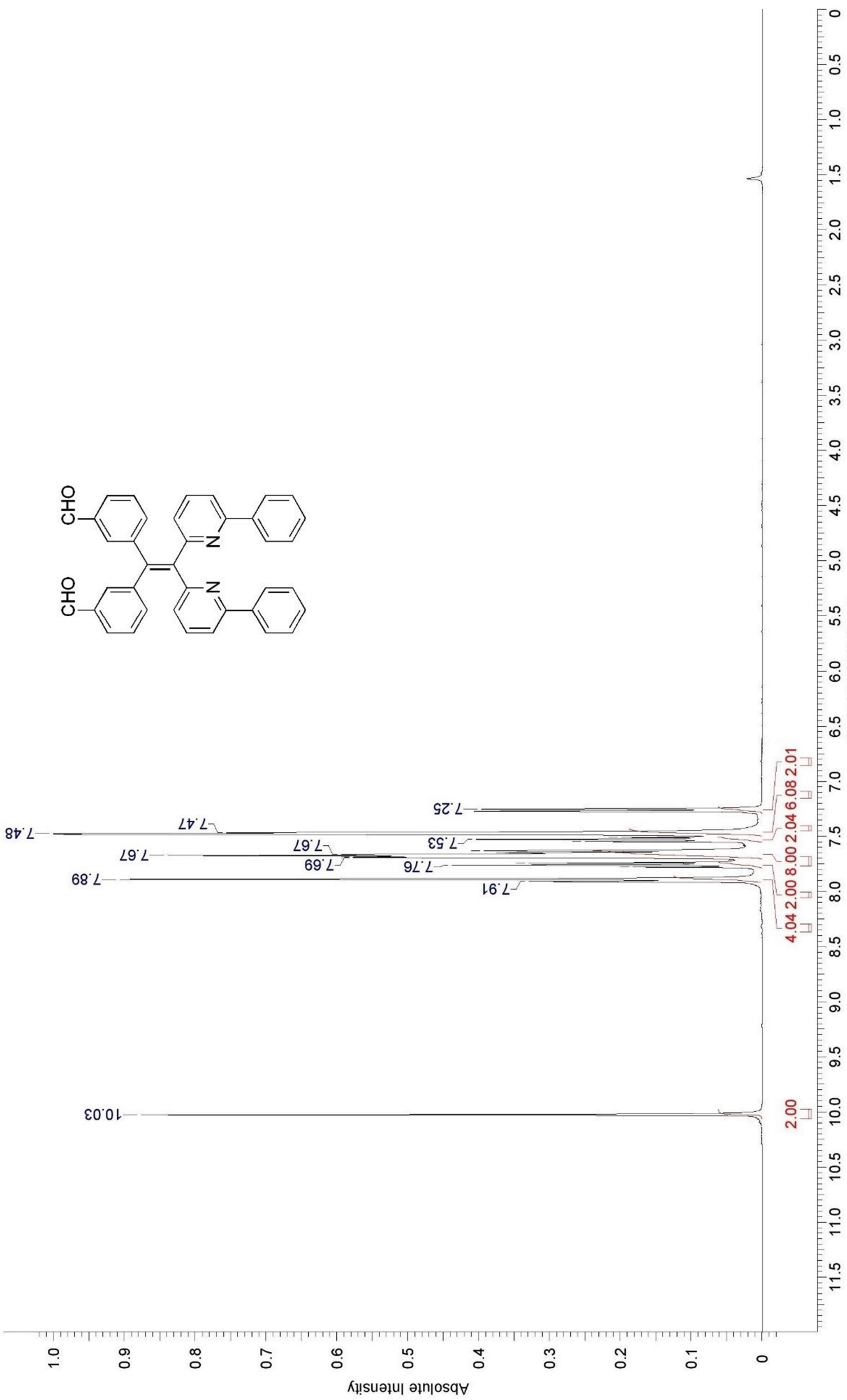
CHO
CHO
N
N
10.03
7.91
7.89
7.76
7.69
7.67
7.67
7.53
7.48
7.47
7.25
2.00
4.04 2.00 8.00 2.04 6.08 2.01
Chemical Shift (ppm)
Absolute Intensity

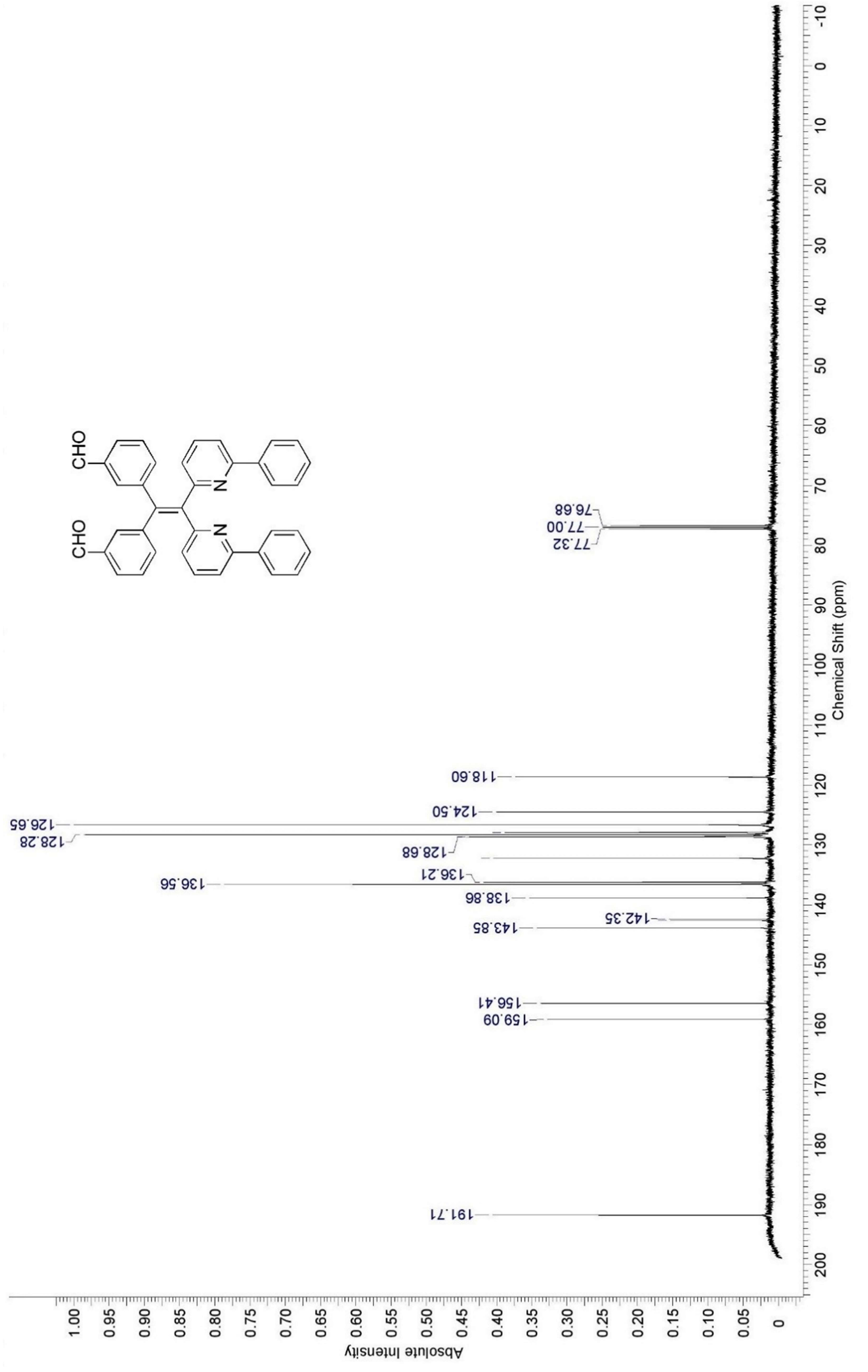
CHO
CHO
N
N
191.71
159.09
156.41
143.85
142.35
138.86
136.56
136.21
128.68
128.28
126.65
124.50
118.60
77.32
77.00
76.68
Chemical Shift (ppm)
Absolute Intensity

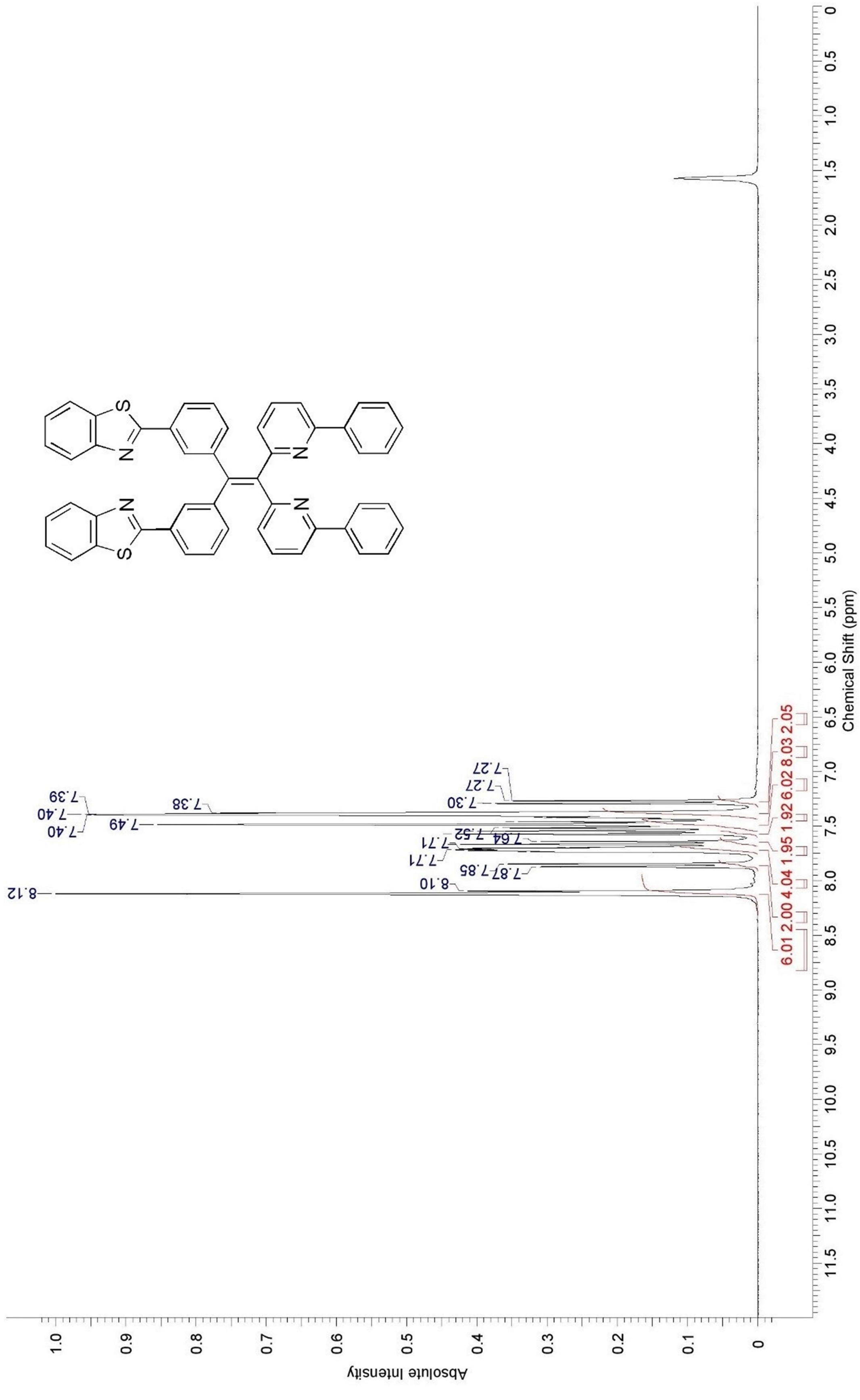

8.12
8.10
7.87
7.85
7.71
7.71
7.64
7.52
7.49
7.40
7.40
7.39
7.38
7.30
7.27
7.27
6.01 2.00 4.04 1.95 1.92 6.02 8.03 2.05
Chemical Shift (ppm)
Absolute Intensity

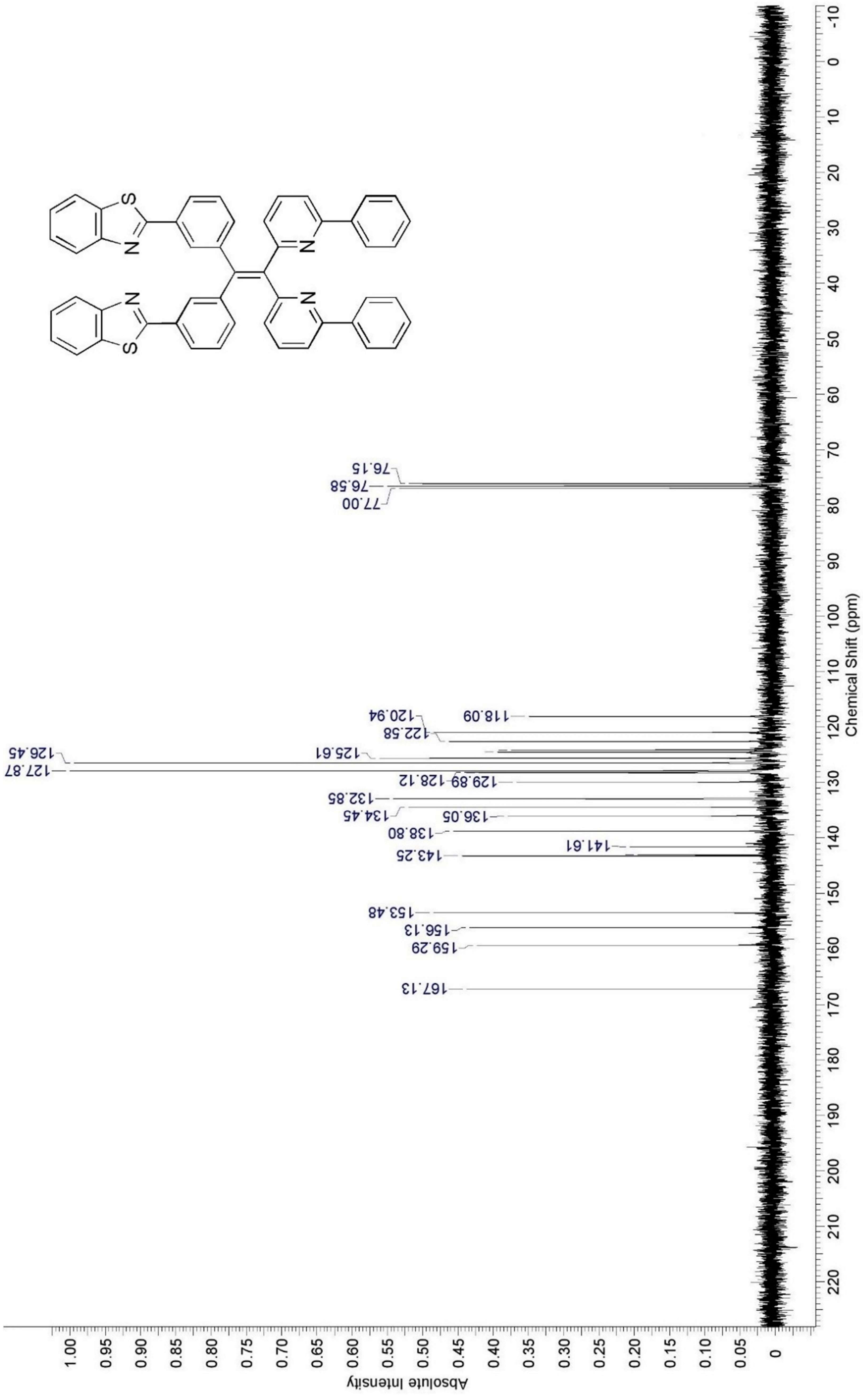

77.00
76.58
76.15
118.09
120.94
122.58
125.61
126.45
127.87
128.12
129.89
132.85
134.45
136.05
138.80
141.61
143.25
153.48
156.13
159.29
167.13
Chemical Shift (ppm)
Absolute Intensity

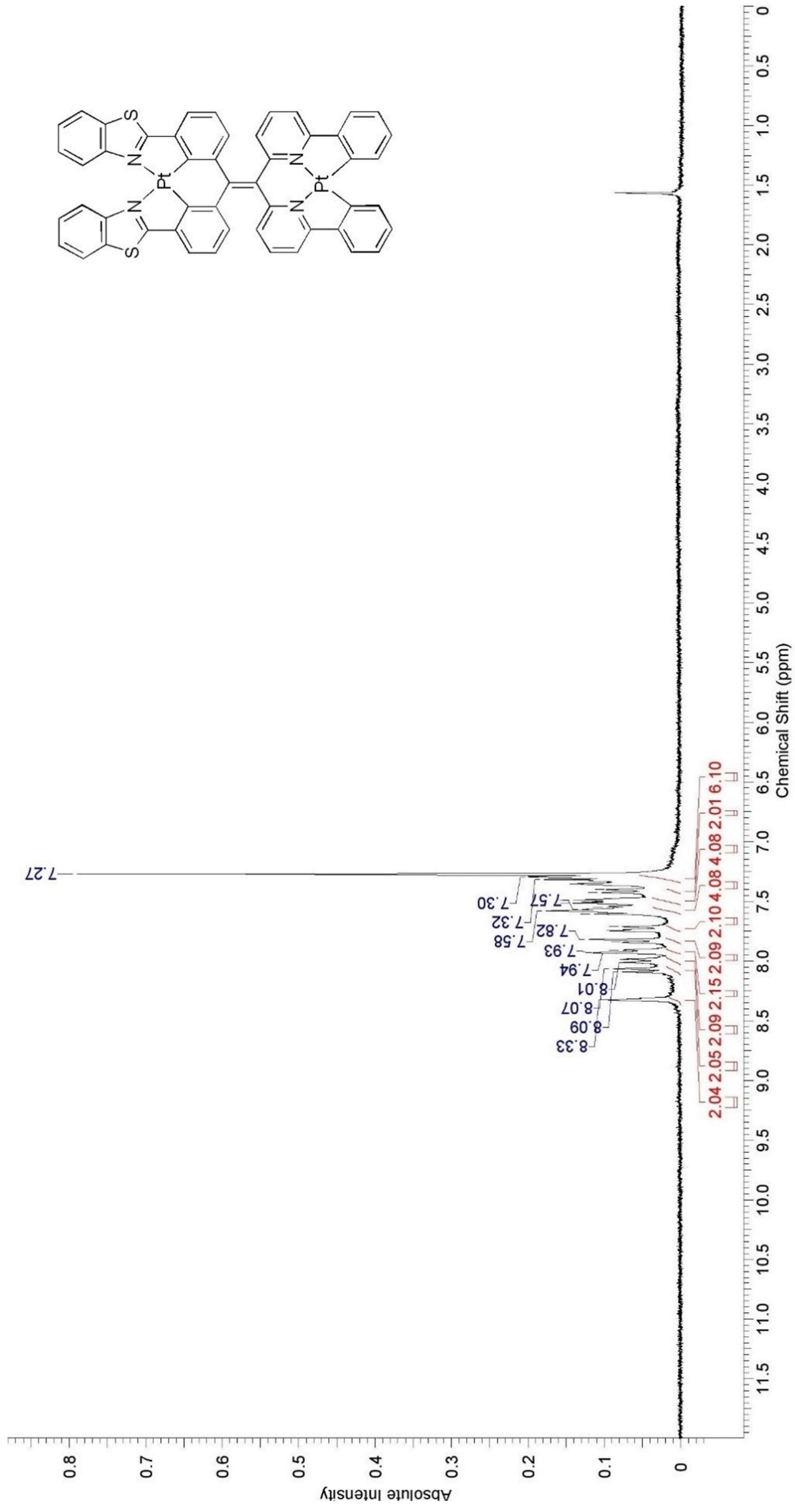

7.27
7.30
7.32
7.57
7.58
7.82
7.93
7.94
8.01
8.07
8.09
8.33
2.04 2.05 2.09 2.15 2.09 2.10 4.08 4.08 2.01 6.10
Chemical Shift (ppm)
Absolute Intensity

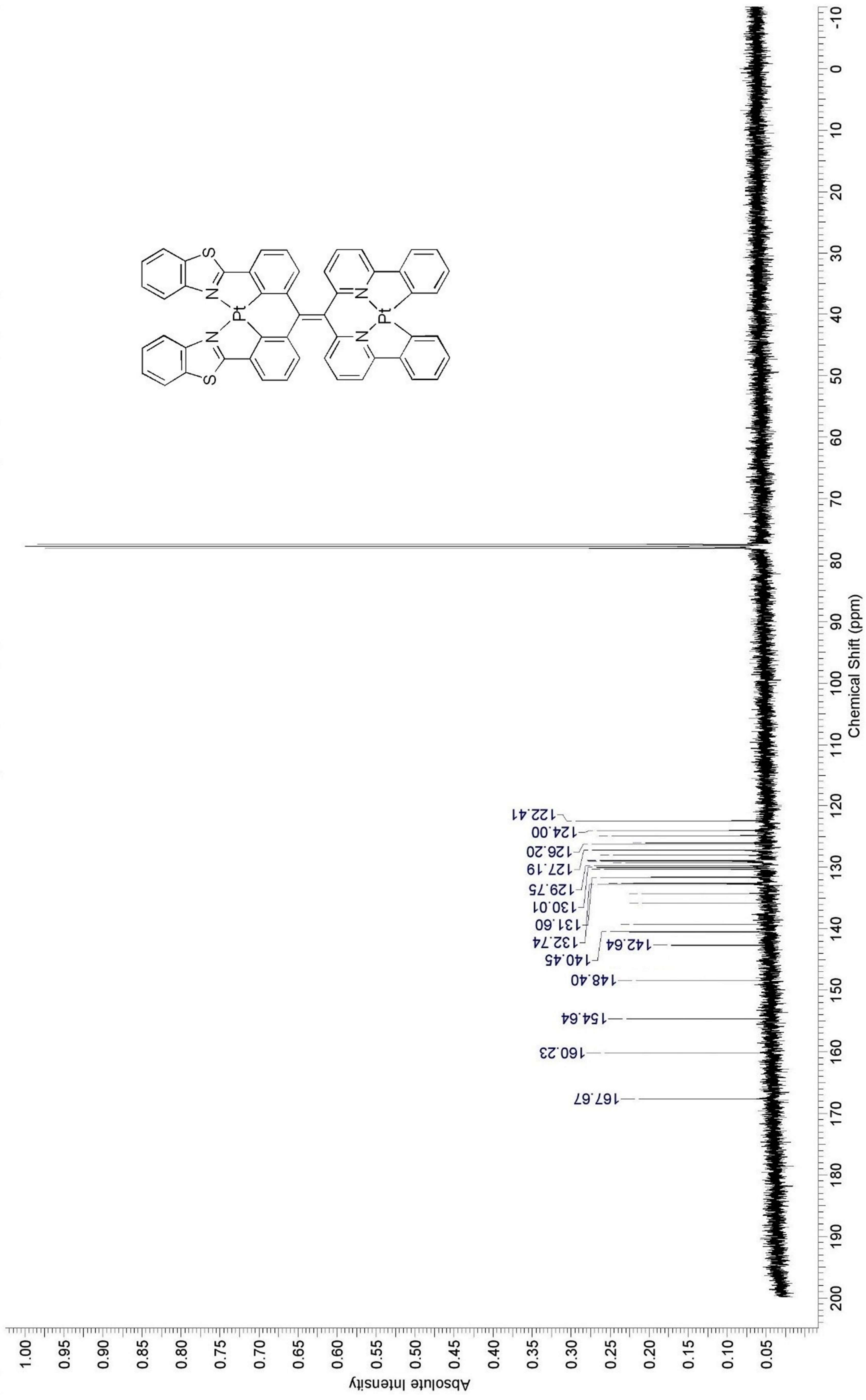
122.41
124.00
126.20
127.19
129.75
130.01
131.60
132.74
140.45
142.64
148.40
154.64
160.23
167.67
Chemical Shift (ppm)
Absolute Intensity
Pt
Pt
N
N
N
N
S
S

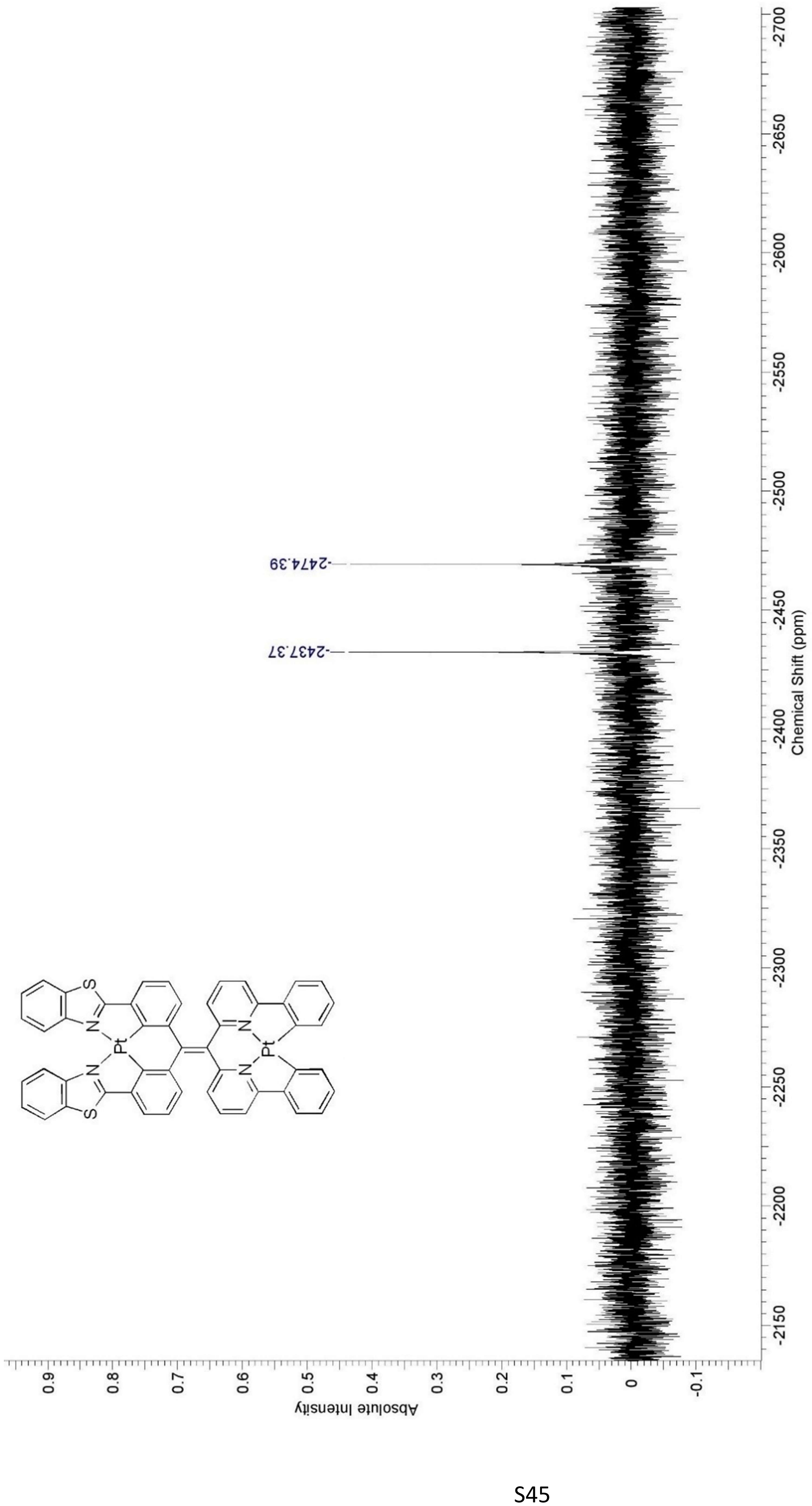
-2474.39
-2437.37
Chemical Shift (ppm)
Absolute Intensity
Pt
Pt
N
N
N
N
S
S

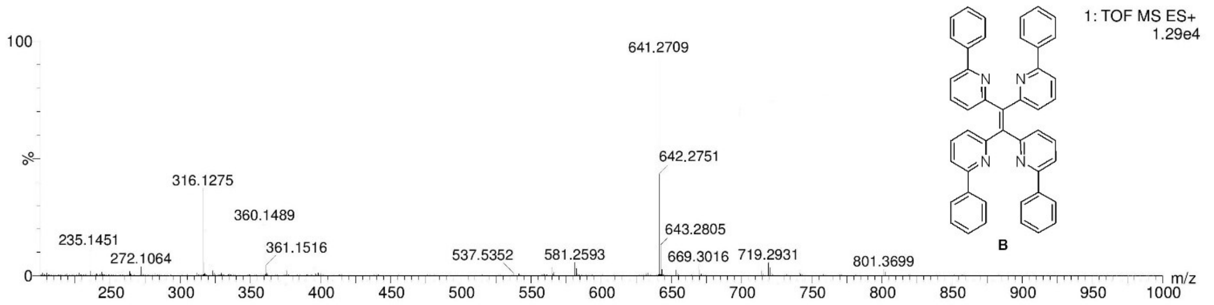
1: TOF MS ES+
1.29e4
641.2709
642.2751
316.1275
360.1489
643.2805
235.1451
361.1516
272.1064
537.5352
581.2593
669.3016
719.2931
801.3699
m/z
B

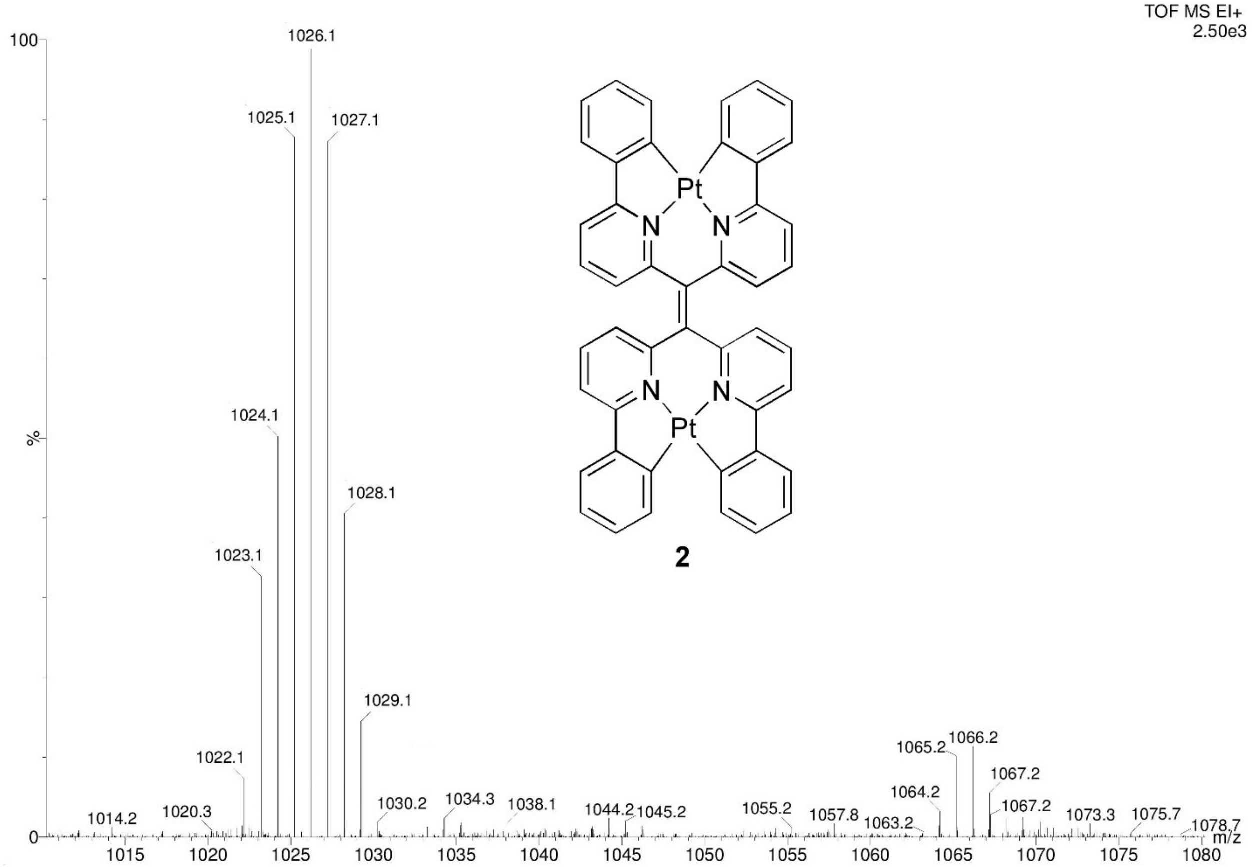
TOF MS EI+
2.50e3
1026.1
1025.1
1027.1
1024.1
1028.1
1023.1
1029.1
1022.1
1065.2
1066.2
1067.2
1064.2
m/z
Pt
N
2

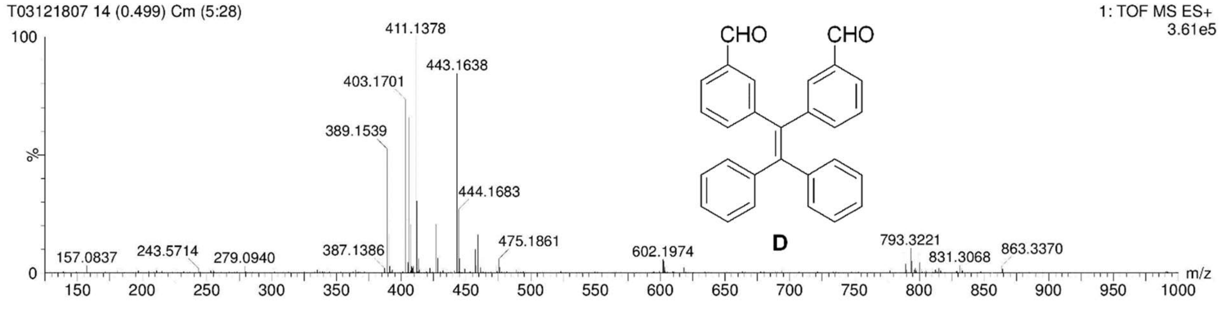
T03121807 14 (0.499) Cm (5:28)
1: TOF MS ES+
3.61e5
411.1378
443.1638
403.1701
389.1539
444.1683
475.1861
157.0837
243.5714
279.0940
387.1386
602.1974
793.3221
831.3068
863.3370
m/z
CHO
D

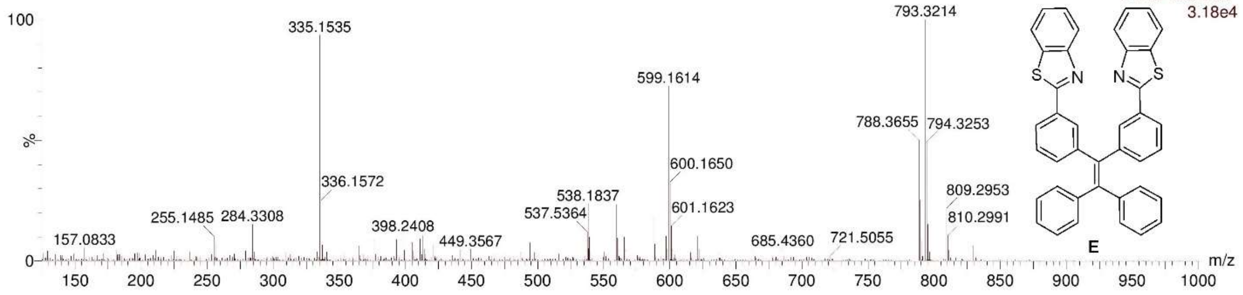

1: TOF MS ES+
3.18e4
100
%
0
335.1535
336.1572
599.1614
600.1650
601.1623
793.3214
788.3655
794.3253
809.2953
810.2991
157.0833
255.1485
284.3308
398.2408
449.3567
537.5364
538.1837
685.4360
721.5055
m/z
150
200
250
300
350
400
450
500
550
600
650
700
750
800
850
900
950
1000
S
N
E

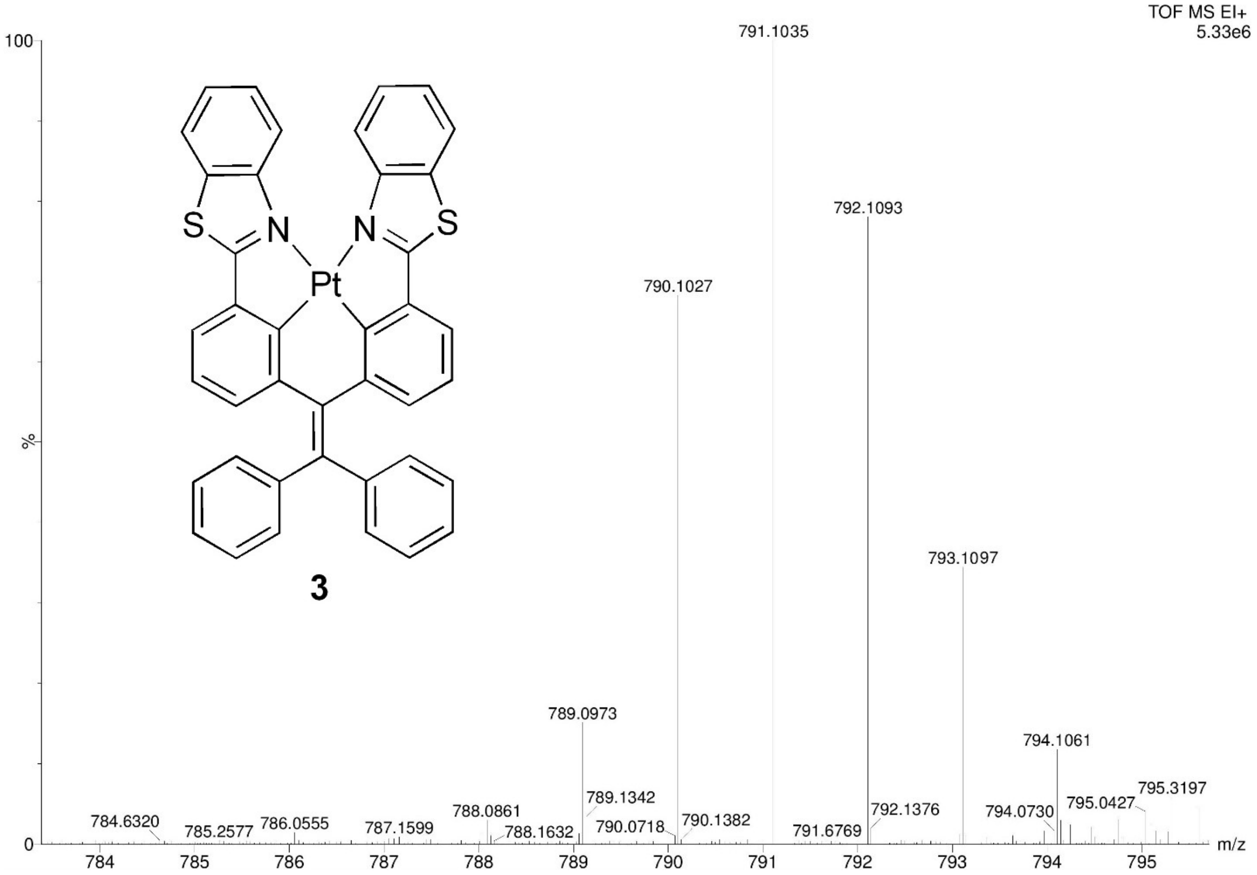

TOF MS EI+
5.33e6
100
%
0
791.1035
792.1093
790.1027
793.1097
789.0973
794.1061
795.3197
784.6320
785.2577
786.0555
787.1599
788.0861
788.1632
789.1342
790.0718
790.1382
791.6769
792.1376
794.0730
795.0427
m/z
784
785
786
787
788
789
790
791
792
793
794
795
S
N
Pt
3

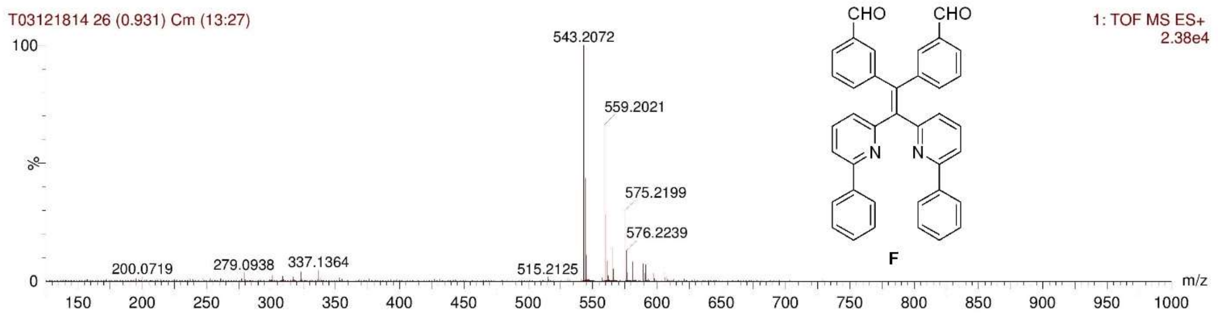
T03121814 26 (0.931) Cm (13:27)
1: TOF MS ES+
2.38e4
543.2072
559.2021
575.2199
576.2239
200.0719
279.0938
337.1364
515.2125
CHO
CHO
N
N
F
%
m/z

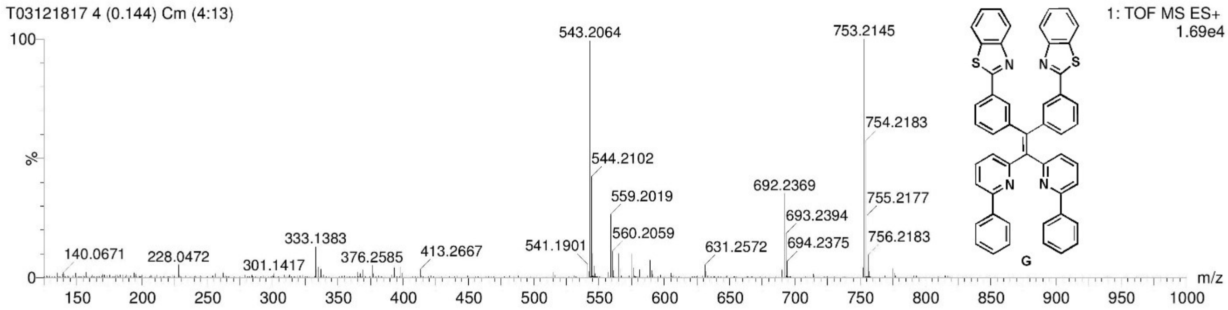
T03121817 4 (0.144) Cm (4:13)
1: TOF MS ES+
1.69e4
543.2064
753.2145
754.2183
544.2102
559.2019
560.2059
692.2369
693.2394
694.2375
755.2177
756.2183
140.0671
228.0472
301.1417
333.1383
376.2585
413.2667
541.1901
631.2572
G
%
m/z

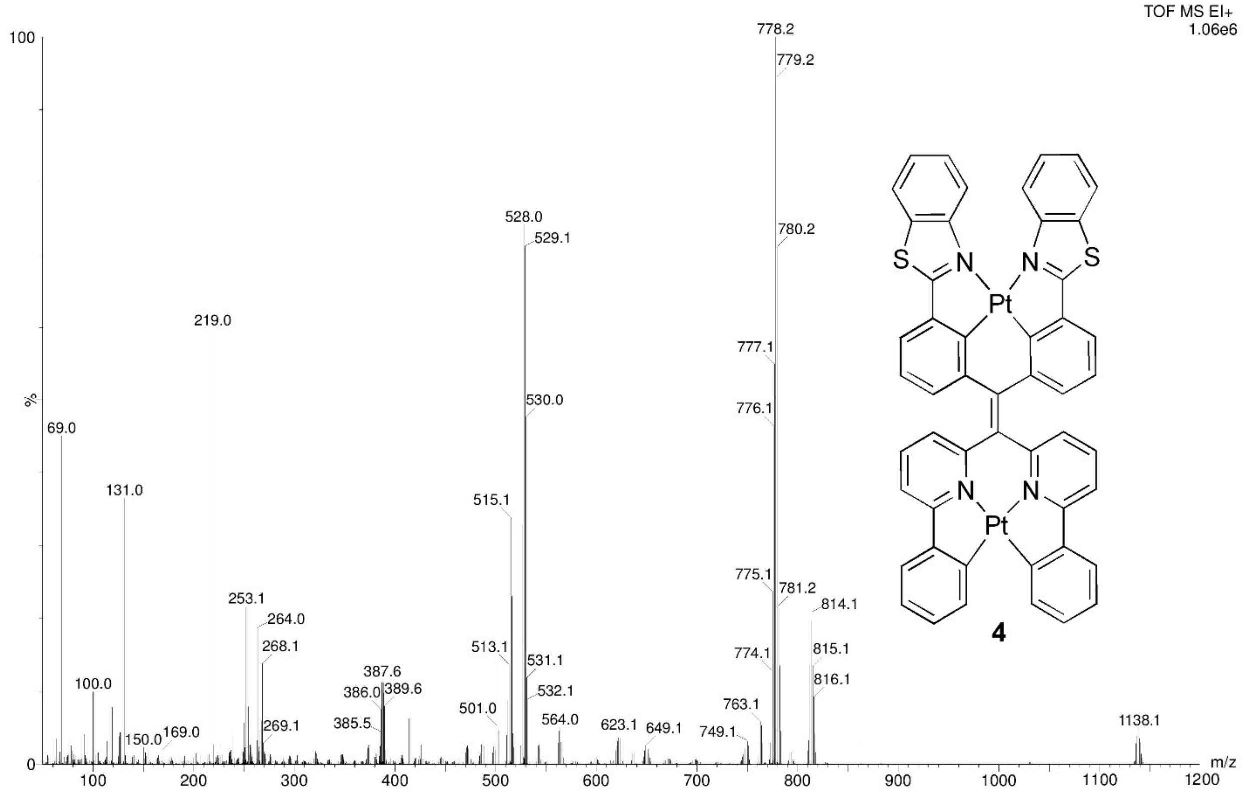
TOF MS EI+
1.06e6
778.2
779.2
780.2
528.0
529.1
219.0
777.1
530.0
776.1
69.0
131.0
515.1
775.1
781.2
814.1
253.1
264.0
268.1
513.1
531.1
774.1
815.1
816.1
387.6
386.0
389.6
532.1
100.0
763.1
501.0
564.0
623.1
649.1
749.1
269.1
385.5
150.0
169.0
1138.1
Pt
Pt
4
%
m/z
